# Near-infrared fluorescent probe for hydrogen sulfide: high-fidelity ferroptosis evaluation *in vivo* during stroke†

**DOI:** 10.1039/d1sc05930k

**Published:** 2022-02-21

**Authors:** Tianyu Liang, Taotao Qiang, Longfang Ren, Fei Cheng, Baoshuai Wang, Mingli Li, Wei Hu, Tony D. James

**Affiliations:** College of Bioresources and Materials Engineering, Shaanxi Collaborative Innovation Center of Industrial Auxiliary Chemistry & Technology, Shaanxi University of Science & Technology Xi'an 710021 China qiangtt515@163.com huwchem@163.com; Department of Chemistry, University of Bath Bath BA27AY UK t.d.james@bath.ac.uk; School of Chemistry and Chemical Engineering, Henan Normal University Xinxiang 453007 China

## Abstract

Ferroptosis is closely associated with cancer, neurodegenerative diseases and ischemia-reperfusion injury and the detection of its pathological process is very important for early disease diagnosis. Fluorescence based sensing technologies have become excellent tools due to the real-time detection of cellular physiological or pathological processes. However, to date the detection of ferroptosis using reducing substances as markers has not been achieved since the reducing substances are not only present at extremely low concentrations during ferroptosis but also play a key role in the further development of ferroptosis. Significantly, sensors for reducing substances usually consume reducing substances, instigating a redox imbalance, which further aggravates the progression of ferroptosis. In this work, a H_2_S triggered and H_2_S releasing near-infrared fluorescent probe (HL-H_2_S) was developed for the high-fidelity *in situ* imaging of ferroptosis. In the imaging process, HL-H_2_S consumes H_2_S and releases carbonyl sulfide, which is then catalyzed by carbonic anhydrase to produce H_2_S. Importantly, this strategy does not intensify ferroptosis since it avoids disruption of the redox homeostasis. Furthermore, using erastin as an inducer for ferroptosis, the observed trends for Fe^2+^, MDA, and GSH, indicate that the introduction of the HL-H_2_S probe does not exacerbate ferroptosis. In contrast, ferroptosis progression was significantly promoted when the release of H_2_S from HL-H_2_S was inhibited using AZ. These results indicate that the H_2_S triggered and H_2_S releasing fluorescent probe did not interfere with the progression of ferroptosis, thus enabling high-fidelity *in situ* imaging of ferroptosis.

## Abstract 2

A H_2_S triggered and H_2_S releasing near-infrared fluorescent probe (HL-H_2_S) was developed. HL-H_2_S does not interfere with the progression of ferroptosis by consuming H_2_S, thus enabling high-fidelity *in situ* imaging of ferroptosis.

## Graphical abstract

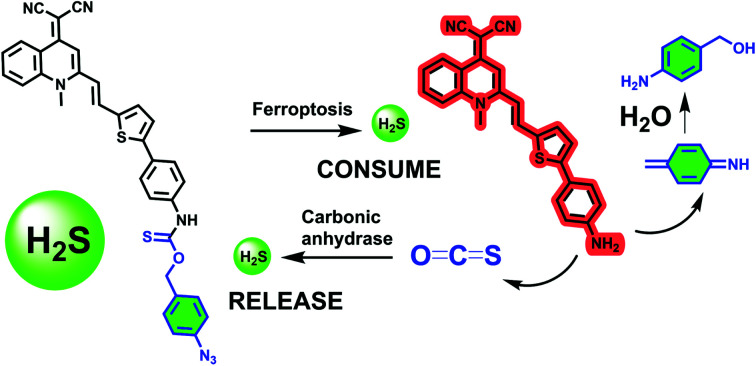

## Introduction

With strong tissue penetration ability, minor cell and tissue damage, and low interference from autofluorescence, near-infrared (NIR) fluorescence imaging is gradually replacing traditional fluorescence imaging as one of the most popular tools for real-time, *in situ*, visual tracking of biomolecules.^1–5^ The recent success of *in situ* tracking of ferroptosis (an iron-dependent oxidative stress) based on NIR fluorescence sensing has provided a basis for the treatment and drug design for neurodegenerative diseases, acute kidney injuries, and malignant tumors.^6,7^ However, the current NIR fluorescent probes used to monitor ferroptosis progression detect oxidizing substances such as lipid ROS and free ferrous ions, but the detection of reducing substances is rarely reported.^6,8,9^ Compared with oxidizing substances, reducing substances such as GSH and GPX4 are considered to be the key biomarkers to directly monitor ferroptosis, because ferroptosis can be regulated by the X_c_^−^/GSH/GPX4 system by controlling the generation of phospholipid hydroperoxides.^10^ Current methods for the detection of antioxidants GSH or GPX4, for example western blotting make it impossible to achieve high sensitivity, *in situ* imaging and real-time tracking.^11,12^ As such the development of probes able to understand cell redox are required in order to understand cell ferroptosis, since the molecular regulation mechanisms of ferroptosis are complicated, and remain to be fully elucidated. In biology, the level of ferroptosis is determined by the simultaneous characterization of multiple targets.^13–15^

As a gaseous signaling molecule, H_2_S plays an important role in human physiological and pathological processes.^16,17^ H_2_S has been defined as an important endogenous neuroprotective agent that exerts protective effects through antioxidant, anti-inflammatory, and anti-apoptotic mechanisms.^18,19^ A significant amount of evidence suggests that endogenous H_2_S is produced from cysteine desulfurization catalyzed by cystathionine γ lyase (CSE) or cystathionine β synthase (CBS).^20,21^ Since ferroptosis inhibits the cystine/glutamate transporter (system x_c_^−^), cysteine uptake is decreased, which leads to H_2_S depletion.^22^ Thus, H_2_S can be considered as a representative reducing substance and as such enables monitoring of the ferroptosis process. However, it is difficult for current NIR fluorescent probes to facilitate *in situ* H_2_S tracking because extremely low concentrations of H_2_S will enhance the progression of ferroptosis.^21,23^ Significantly, traditional sensors for reducing substances require the consumption of the reducing substance, leading to a redox imbalance, which further aggravates the progression of ferroptosis.^24–26^

Aiming to address these challenges, we have developed a benzyl thiocarbamate with an azide as the recognition site for H_2_S. Carbonyl sulfide (COS) is released through 1,6-elimination induced by H_2_S attack, which in turn releases H_2_S on catalysis by carbonic anhydrase (CA).^27–30^ This H_2_S triggered and H_2_S releasing fluorescent probe has the potential to break through the current bottleneck and thus increase the accuracy in detecting ferroptosis. From our experimental results, the progression of erastin induced ferroptosis in cells with and without HL-H_2_S were not significantly different. In contrast, the progression of ferroptosis was significantly promoted when the H_2_S release from HL-H_2_S was inhibited using AZ. Our results indicate that HL-H_2_S with both H_2_S triggered and H_2_S releasing mechanisms does not induced ferroptosis. Therefore, our probe is capable of *in situ* high-fidelity ferroptosis analysis, making it a reliable tool for the comprehensive and accurate understanding of ferroptosis progression.

## Results and discussion

### Design strategy and synthesis of fluorescent probe HL-H_2_S

**Scheme 1:**
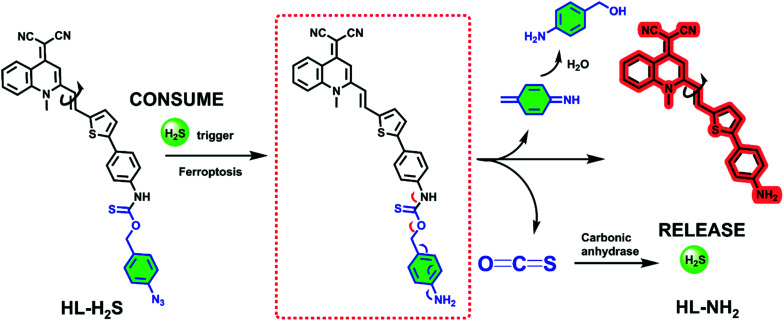
H_2_S triggered and H_2_S releasing probe for high-fidelity ferroptosis evaluation and mechanistic details for COS/H_2_S release by HL-H_2_S.

The response mechanism of the H_2_S triggered and H_2_S releasing probe HL-H_2_S are shown in Scheme 1. With NIR emission, HL-H_2_S is endowed with good tissue penetration ability, which results in minor cell and tissue damage, and low interference from autofluorescence.^31^ An azido benzene was used as the H_2_S recognition site^32,33^ which was linked to the fluorophore using a thiocarbamate (H_2_S precursor). When HL-H_2_S responds to H_2_S, COS is released through 1,6-elimination, which is then catalyzed by CA to release H_2_S. Thus, a novel H_2_S triggered and H_2_S releasing system results. Most notably, quinolinemalonitrile is linked to the thiophene in HL-NH_2_ by σ-bonds, so molecular rotation of its structure will be regulated by viscosity. This enabled us to accurately detect the ferroptosis process, because cell viscosity increases during ferroptosis.^6,8,34^HL-H_2_S could be used to detect the ferroptosis of cells of high viscosity, but there was no significant change in normal cells.^6,35^ In a high-viscosity environment, the rotation of HL-NH_2_ is hindered, resulting in an increase of quantum yield from 0.01 to 0.67, as shown in Table S1.† As such the sensitivity of HL-NH_2_, is enhanced in a viscous environment facilitating accurate detection of ferroptosis. The detailed synthetic procedures and characterization data for probe HL-H_2_S are described in Scheme S1.† The response mechanism of the probe was confirmed by monitoring the ^1^H NMR and verifying the molecular weight before and after reaction using HR-MS (Fig. S1 and S2†). When HL-H_2_S reacts with NaHS (H_2_S donor), the signal at *δ* 11.4 (H_a_) disappears, and a molecular ion peak *m*/*z* 429.11169 using HR-MS confirms the formation of HL-NH_2_ (*m*/*z* 429.11444, [M + Na]^+^).

### Photophysical properties of HL-NH_2_

With probe HL-H_2_S in hand, the photophysical properties of the fluorophore HL-NH_2_ in different solvents were systematically investigated to optimize the detection system. Fig. S3a and b† show the absorption and fluorescence spectra of HL-NH_2_, respectively, and the photophysical parameters are given in Table S1.† In glycerol, the fluorescence intensity of HL-NH_2_ was the highest at 646 nm (quantum yield = 0.67), the molar absorptivity reached 0.64 × 10^4^ M^−1^ cm^−1^ (*λ*_abs_ = 430 nm), and the Stokes shift was 216 nm. Therefore, light scattering due to excitation was avoided, and the resolution and accuracy of the probe improved. According to Fig. S3d,† the fluorescence intensity of HL-NH_2_ was highest in glycerol, indicating that the fluorescence intensity of HL-NH_2_ is affected by viscosity. The sensitivity of HL-NH_2_ to polarity was also analyzed. As shown in Fig. S3c,† the fluorescence intensity of HL-NH_2_ does not change as the dielectric constant of the solvent increases, indicating that fluorescence emission of HL-NH_2_ is not affected by the polarity of the solvent. According to the literature,^36,37^ quinolinemalonitrile derivates exhibit aggregation-induced emission (AIE). The AIE performance of HL-NH_2_ was investigated in a mixture of tetrahydrofuran (THF) and water, where THF was a good solvent for HL-NH_2_ and water was a poor solvent. As shown in Fig. S4a,† the absorption intensity of HL-NH_2_ remains low without significant changes as the water content (*f*_w_) decreased from 100% to 70%. As *f*_w_ drops below 70%, the absorption intensity of HL-NH_2_ increases abruptly at 450 nm, which may be due to the scattering effect of the aggregates generated *in situ*.^38^ Unlike absorption intensity, the fluorescence intensity of HL-NH_2_ was negligible at 0% and 100% *f*_w_, and reaches a maximum at 40% (Fig. S4b–d†), which is different from conventional AIE luminescence.^39^ We believe that the reason for the difference observed for our system and traditional AIE fluorophores based on quinolinemalonitrile are due to the primary amino group of HL-NH_2_ exhibiting enhanced solubility is PBS through hydrogen bonding with water, which results in reduced AIE.^37^ The response of HL-NH_2_ to viscosity was also evaluated. As shown in Fig. S5a–c,† the fluorescence intensity of HL-NH_2_ gradually increases as the viscosity of the solution is increased from 1.1 cP (0 vol% glycerol) to 1410 cP (99 vol% glycerol) by increasing the proportion of glycerol. According to Fig. S5b and c,† two different linear relationships emerge for volume fraction ranges from 0–40% and 50–99%. For each viscosity range the linear correlation coefficient between the fluorescence intensity of the probe and the viscosity are 0.996, indicating that the probe HL-H_2_S can quantify the viscosity of a target solution. Finally, the aqueous solubility of the probe HL-H_2_S was investigated. The probe was soluble in a water solution at a concentration of 20 μM and exhibited a good linear relationship between solubility and concentration (*R*^2^ = 0.974). Therefore, the solubility of HL-H_2_S is sufficient for *in vivo* and *in vitro* experiments (Fig. S6†).

### Spectroscopic response of HL-H_2_S to H_2_S

**Fig. 1:**
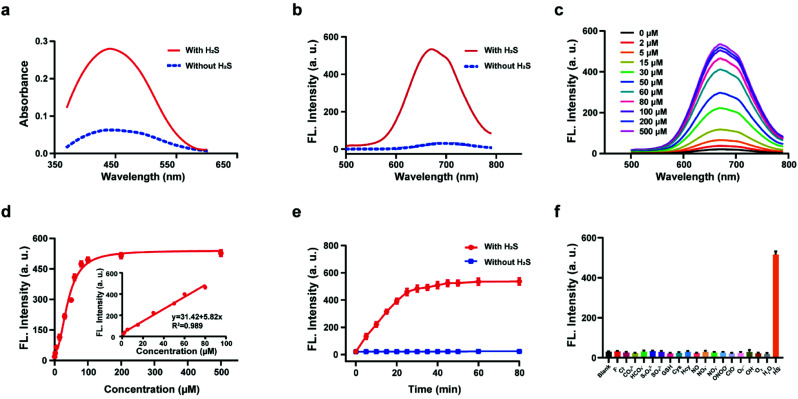
Absorption spectra (a) and fluorescence spectra (b) of probe HL-H_2_S with H_2_S (from NaHS) concentration (100 μM) with H_2_S (solid line) and without H_2_S (dotted line). (c) Fluorescence spectra of HL-H_2_S with the increasing H_2_S concentration (0–500 μM). (d) Calibration curve for the determination of H_2_S (inset linear response at lower H_2_S concentrations). (e) Time dependent fluorescence intensity of HL-H_2_S in the absence and presence of H_2_S (100 μM). (f) Response of HL-H_2_S to 200 μM different interferents (HL-H_2_S alone (blank)); F^−^, Cl^−^, CO_3_^2−^, HCO_3_^−^, S_2_O_3_^2−^, SO_4_^2−^, GSH, Cys, Hcy, NO, NO_3_^−^, NO_2_^−^, ONOO^−^, ClO^−^, O_2_˙^−^, ˙OH, ^1^O_2_, H_2_O_2_, HS^−^, respectively. For all experiments the concentration of HL-H_2_S was 10 μM. *λ*_ex_ = 450 nm, in PBS (pH = 7.4, containing 2% DMSO and 80% glycerol). In (d–f), data represent the mean of three replicates and the error bars indicate the SD.

The response of the probe HL-H_2_S to H_2_S (from NaHS) was investigated in phosphate buffer solution (PBS, pH = 7.4, containing 80% glycerol and 2% DMSO). The addition of 100 μM H_2_S resulted in a significant increase in the spectral intensity of both the UV and fluorescence (Fig. 1a and b), indicating excellent response of HL-H_2_S towards H_2_S. The fluorescence titration experiments (Fig. 1c) indicated that as the H_2_S concentration increased from 0 to 500 μM, the fluorescence intensity of the solution (*λ*_em_ = 670 nm) increased by around 25-fold before reaching a plateau at a H_2_S concentration of 100 μM (Fig. 1d). While the fluorescence intensity exhibited a good linear relationship with concentrations of H_2_S over a range from 1–80 μM (Fig. 1d inset). The detection limit was calculated to be 1.3 nM. The above experiments indicated that HL-H_2_S exhibits a dual response capability for viscosity and H_2_S. The response of HL-H_2_S was then analyzed in terms of kinetics, pH stability, and selectivity to further investigate the performance towards H_2_S. Kinetic analysis indicated that the reaction between HL-H_2_S and H_2_S (100 μM) was completed within 40 min, indicating that HL-H_2_S is capable of quickly identifying H_2_S (Fig. 1e). The effect of different pH environments on HL-H_2_S and its response to H_2_S were then evaluated. As shown in Fig. S7,† over a pH range from 5.0 to 8.5, the fluorescence intensity of HL-H_2_S does not change significantly regardless of the presence of H_2_S, indicating that the response of HL-H_2_S to H_2_S is not affected by changes in environmental pH. Finally, the influence of reactive oxygen (ClO^−^, O_2_˙^−^, ˙OH, ^1^O_2_, and H_2_O_2_), reactive nitrogen (NO_2_^−^, NO_3_^−^, NO, and ONOO^−^), reactive sulfur (GSH, Cys, Hcy, S_2_O_3_^2−^, SO_4_^2−^and HS^−^) and other anions (F^−^, Cl^−^, CO_3_^2−^, HCO_3_^−^) on the response of HL-H_2_S were evaluated. As shown in Fig. 1f, these interfering species hardly affect the fluorescence intensity of the system, while the introduction of H_2_S significantly enhanced the fluorescence intensity of the system, indicating that HL-H_2_S exhibits a high selectivity for H_2_S.

### *In vitro* H_2_S release of HL-H_2_S

**Fig. 2:**
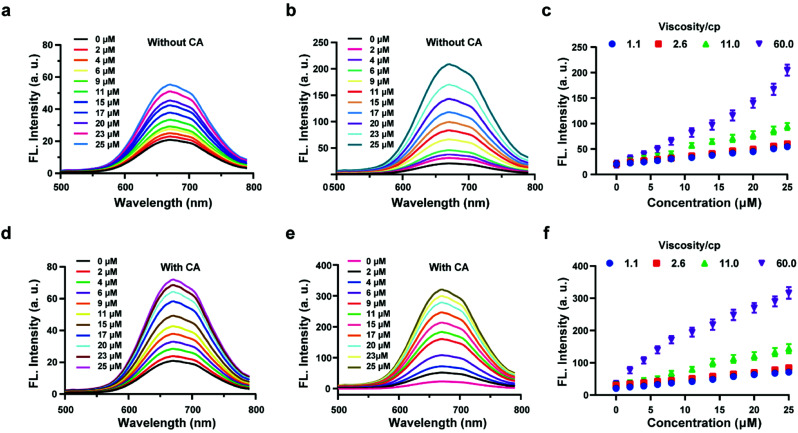
Probe HL-H_2_S (10 μM) with increasing H_2_S concentrations (0–25 μM) in PBS (pH = 7.4, containing 2% DMSO). Fluorescence spectra of HL-H_2_S (10 μM) in the absence of carbonic anhydrases (CA) with the increasing H_2_S concentration (0–25 μM) in the absence (a) and presence (b) of glycerol (80% in volume, viscosity: 60.0 cP). (c) Dose-dependent titrations between HL-H_2_S and H_2_S concentrations (0–25 μM) with different glycerol level (1.1, 2.6, 11.0 and 66.0 cP). Fluorescence spectra of HL-H_2_S (10 μM) in the presence of carbonic anhydrases (CA) with the increasing H_2_S concentration (0–25 μM) in the absence (d) and presence (e) of glycerol (80% in volume, viscosity: 60.0 cP). (f) Dose-dependent titrations between HL-H_2_S and H_2_S concentrations (0–25 μM) with different glycerol level (1.1, 2.6, 11.0 and 66.0 cP). H_2_S concentration (0, 2, 4, 6, 9, 11, 15, 17, 20, 23 and 25 μM); *λ*_ex_ = 450 nm. In c and f, data represent the mean of three replicates and the error bars indicate the SD.

The above experiments confirmed that the probe HL-H_2_S is capable of H_2_S-specific recognition in a high-viscosity environment. Next, ferroptosis was simulated *in vitro* and the detection and release capacity of the probe for H_2_S was evaluated to facilitate the accurate detection of ferroptosis using the probe. CA has been reported to catalyze the conversion of COS, to produce H_2_S.^27,28^ As shown by Fig. 2a, in the absence of CA and glycerol, a *ca.* ∼3-fold enhancement of the fluorescence with increasing H_2_S concentration was achieved. However, the fluorescence increases 10-fold as the glycerol content increases to 80% (viscosity: 60 cP), and the effect of environmental viscosity on the response of HL-H_2_S to H_2_S is significant (Fig. 2b and c). Compared to the absence of CA, the sensitivity of HL-H_2_S to H_2_S is slightly enhanced in the presence of CA (Fig. 2d). Importantly, Fig. 2e and f show that the fluorescence intensity increases 15-fold with addition of H_2_S, which was attributed to the release of H_2_S from HL-H_2_S following reaction with H_2_S. To verify this conjecture, acetazolamide (AZ), an inhibitor of CA,^40^ was used to regulate the release of H_2_S. Fig. S8 a and b† shows that the fluorescence intensity of the solution with AZ and CA is almost identical to that of the solution without AZ or CA, and both are lower than that of the solution with CA, indicating that under these conditions HL-H_2_S can be catalyzed by CA to produce H_2_S. To verify the conversion of decomposed HL-H_2_S to H_2_S in the presence of CA, the well-known methylene blue (MB) method^29^ was used to measure the generation of H_2_S under the above conditions. The results indicated that the changes of H_2_S monitored using MB were consistent with the fluorescence results obtained for the probe, indicating that under these conditions HL-H_2_S can be catalyzed by CA to produce H_2_S (Fig. S8c†), which confirms that HL-H_2_S can release H_2_S in the presence of CA. Fig. S8d† is the calibration curve between concentrations of H_2_S (0–20 μM) and absorption intensity at 670 nm.

### Imaging of H_2_S and cytoplasmic viscosity in living cells with HL-H_2_S

After confirming the H_2_S response and H_2_S release capacity of HL-H_2_S for *in vitro* experiments, we anticipated that HL-H_2_S can keep the level of cell ferroptosis form being aggravated during ferroptosis monitoring, which excludes the possibility of HL-H_2_S-induced ferroptosis, thus enabling high-fidelity imaging and analysis of ferroptosis. Before performing cellular confocal imaging, the biocompatibility of HL-H_2_S was evaluated. As shown in Fig. S9,† the survival rate of PC12 cells is over 90% after 24 h coincubation with HL-H_2_S at different concentrations (0–30 μM) according to MTT assay, indicating that HL-H_2_S has good biocompatibility and is suitable for *in situ* imaging analysis. The dual response of HL-H_2_S to H_2_S and viscosity was then evaluated at the cellular level. Intracellular H_2_S content and viscosity levels were regulated using NaHS (10 μM) to release H_2_S, viscosity inducer monensin^41^ (10 μM), and different cell incubation temperatures (25 °C and 4 °C) since lowering the temperature increases the intracellular viscosity.^42,43^ As shown in Fig. S10a and b,† the red channel fluorescence intensity of the groups with NaHS or monensin were slightly higher than those of the control group. However, the red channel fluorescence intensity of the group with NaHS and monensin was significantly higher than that of the first three groups. In addition, as the incubation temperature decreased, the red channel fluorescence intensity in the group with NaHS increased significantly, while that of the group without NaHS remained unchanged (Fig. S10c and d†). The above experiments indicate that HL-H_2_S has dual response to viscosity and H_2_S at the cellular level. The ability of HL-H_2_S to detect exogenous and endogenous H_2_S was then assessed. As shown in Fig. S11,† PC12 cells were incubated with HL-H_2_S (10 μM) for 10 min to ensure complete entry into the cells. Then the cells were incubated for 40 min with monensin (10 μM), sulfhydryl scavenger *N*-ethylmaleimide^44^ (NEM, 0.5 mM), and different concentrations of NaHS (H_2_S donor) (0, 5, 10, or 20 μM). The experimental results indicated that the red channel fluorescence intensity gradually increased with an increase of H_2_S concentration (Fig. S11a and b†), indicating that HL-H_2_S could detect exogenous H_2_S. It should be noted that the fluorescence intensity of the groups with NEM dropped significantly compared to the control group, which was due to the removal of endogenous H_2_S by NEM. Therefore, HL-H_2_S could detect endogenous H_2_S. CA activity was modulated using AZ to illustrate the H_2_S releasing capacity of the probe. According to the results shown in Fig. S12,† the red channel fluorescence intensity of the group with AZ decreases significantly compared to the groups without AZ. Meanwhile, since exogenous H_2_S is present in cells, regardless of whether the cells were co-incubated with AZ, the result is consistent with Fig. S8.† However, the fluorescence intensity difference is negligible between the groups with and without NEM. The above experiments show that HL-H_2_S is capable of H_2_S response and release at the cellular level.

### Imaging of ferroptosis during OGD/R

**Fig. 3:**
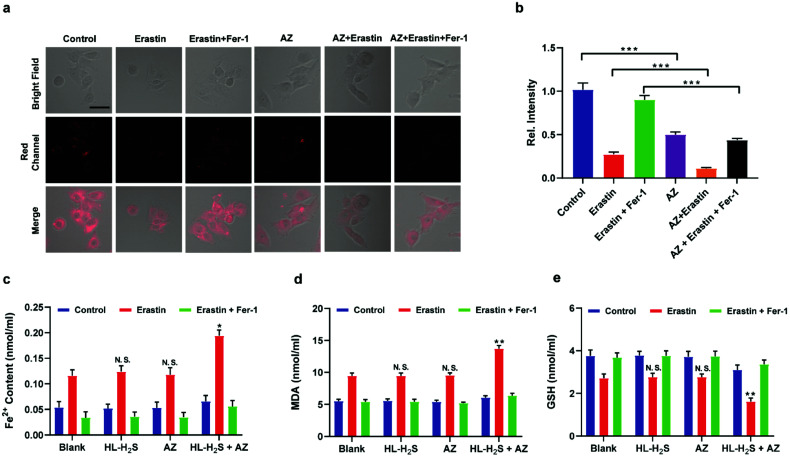
Confocal images of H_2_S in ferroptosis. (a) PC12 cells under different conditions: control group (only HL-H_2_S, 10 μM); HL-H_2_S (10 μM) + erastin (10 μM); HL-H_2_S (10 μM) + erastin (10 μM) + Fer-1 (5 μM). (b) Histograms of average fluorescent intensities in (a) Difference was analyzed by one-way ANOVA. ***p < 0.001. *λ*_ex_ = 450 nm, *λ*_em_ = 630–710 nm. Scale bars: 40 μm. (c) PC12 cells under different conditions: control group (HL-H_2_S (10 μM) + acetazolamide (AZ, 50 μM)); HL-H_2_S (10 μM) + AZ (50 μM) + erastin (10 μM); HL-H_2_S (10 μM) + AZ (50 μM) + erastin (10 μM) + Fer-1 (5 μM). (d) Histograms of average fluorescent intensities in (c); (e) the content of Fe^2+^. (f) The content of MDA. (g) The content of GSH. For the fluorescence change: data are presented as the mean ± SD (control: *n* = 44 cells from three cultures; HL-H_2_S + erastin: *n* = 43 cells from three cultures; HL-H_2_S + erastin + Fer-1: *n* = 33 cells from three cultures; HL-H_2_S + AZ: *n* = 40 cells from three cultures; HL-H_2_S + AZ + erastin: *n* = 35 cells from three cultures; HL-H_2_S + AZ + erastin + Fer-1: *n* = 33 cells from three cultures). *p < 0.05, **P < 0.01 *vs.* blank. In (b) and (d–g), the error bars indicate the SD.

**Fig. 4:**
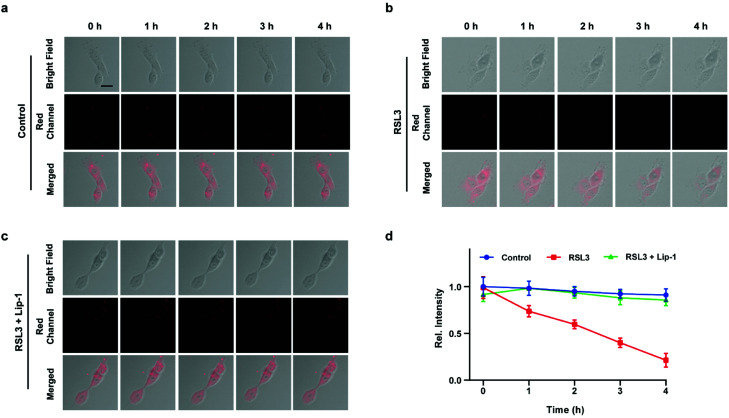
Confocal images of PC12 cells for: (a) control (only HL-H_2_S), (b) RSL3 (1.0 μM, ferroptosis inducer), and (c) RSL3 + Lip-1 (0.5 μM, ferroptosis inhibitor), respectively. Cells were stained with HL-H_2_S (10.0 μM) for 30 min before imaging. (d) Histograms of average fluorescence intensity of HL-H_2_S in control (blue line), RSL3 (red line) and RSL3 + Lip-1 (green line) at different times (0, 1, 2, 3, 4 h). For the fluorescence change: control group: *n* = 43 cells from three cultures; RSL3 group: *n* = 41 cells from three cultures; RSL3 + Lip-1 group: *n* = 51 cells from three cultures. *λ*_ex_ = 450 nm, *λ*_em_ = 630–710 nm. Scale bars: 20 μm. In (d), data represent the mean of three replicates and the error bars indicate the SD.

Inspired by the above experiments, the high-fidelity response of the H_2_S triggered and H_2_S releasing probe HL-H_2_S to ferroptosis was evaluated. As shown in Fig. 3a and b, a ferroptosis model was built by incubating PC12 cells with the ferroptosis inducer erastin,^45^ in which the red channel fluorescence intensity decreases significant compared to the control group, indicating that endogenous H_2_S is consumed in the ferroptosis process. Meanwhile, the addition of ferroptosis inhibitor ferrostatin-1 (Fer-1)^45^ resulted in a significant increase of red channel fluorescence intensity, indicating that HL-H_2_S is capable of the *in situ* imaging of ferroptosis. Given that Fe^2+^, MDA, and GSH levels are common indicators of ferroptosis progression, the simultaneous increase in Fe^2+^ and MDA and decrease in GSH indicate that a ferroptosis model was successfully constructed^46,47^ (Fig. 3c–e, blank group). Importantly, the Fe^2+^, MDA, and GSH levels were not significantly different between cells with and without HL-H_2_S (As shown in Fig. 3c–e, blank group *vs.*HL-H_2_S group). In addition, regardless of whether the cells undergo ferroptosis, the red channel fluorescence intensity was significantly decreased in cells where H_2_S release was inhibited from HL-H_2_S by AZ (Fig. 3a and b). While Fe^2+^ and MDA levels increased and the GSH level decreased significantly compared to the groups without HL-H_2_S (as shown in Fig. 3c–e, blank group *vs.*HL-H_2_S + AZ group). Moreover, there is no change of Fe, MDA, or GSH content for the AZ only control experiment compared to the control or probe alone, further indicating that AZ has no effect on the degree of cell ferroptosis, and only inhibits the ability of the probe to release H_2_S by inhibiting the activity of CA (as shown in Fig. 3c–e, blank group *vs.* AZ group). These results indicated that the H_2_S triggered and H_2_S releasing fluorescent probe HL-H_2_S maintained the progression of ferroptosis, thus enabling high-fidelity *in situ* imaging of ferroptosis. To further evaluate the capacity of HL-H_2_S for real time monitoring of ferroptosis, confocal imaging of PC12 cells stained with HL-H_2_S were recorded at different times (0, 1, 2, 3, 4 h) under different cell culture conditions including control, RSL3, and RSL3 + liproxstatin-1 (Lip-1). As shown in Fig. 4, the fluorescence of cells with RSL3 (ferroptosis inducer) decreased gradually with time. However, the fluorescence for control cells (only HL-H_2_S) and those pre-treated with Lip-1 (ferroptosis inhibitor^48^) remained constant. Thus, fluorescence intensity change of HL-H_2_S could be used to monitor RSL3-induced ferroptosis in live cells.

**Fig. 5:**
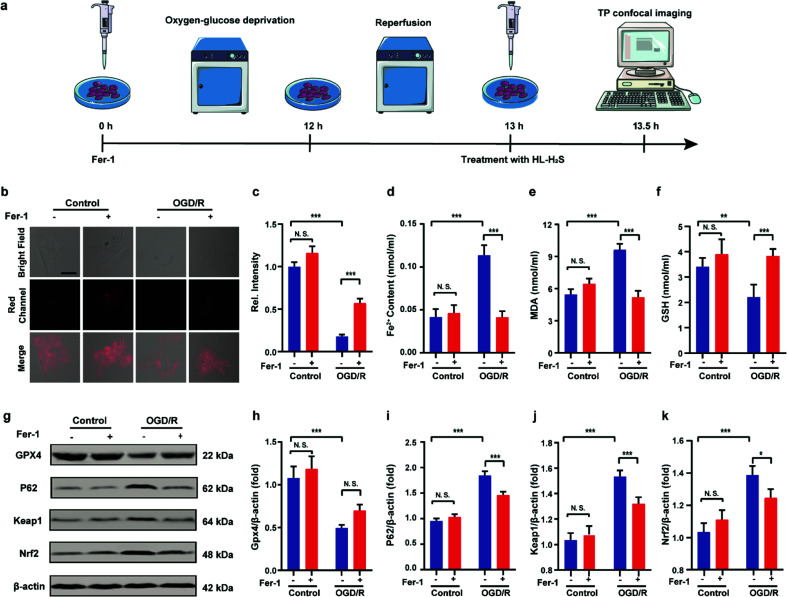
Confocal images of H_2_S in OGD/R. (a) Schematic illustration of oxygen glucose deprivation/reoxygenation (OGD/R) modeling method and probe treatment. (b) PC12 cells using OGD/R models with untreated cells, Fer-1 treated cells, OGD/R treated cells and Fer-1 treated cells during OGD/R. (c) Histograms of average fluorescent intensities in (b); for the fluorescence change: for untreated cells: *n* = 48 cells from three cultures; for Fer-1 treated cells: *n* = 61 cells from three cultures; for OGD/R treated cells: *n* = 44 cells from three cultures; for Fer-1 treated cells during OGD/R: *n* = 51 cells from three cultures. (d) The content of Fe^2+^; (e) the content of MDA; (f) the content of GSH. (g–k) Western blotting analysis of GPX4, p62, Keap1, and Nrf2 in OGD/R model pre-treated with untreated cells, Fer-1 treated cells, OGD/R treated cells and Fer-1 treated cells during OGD/R. Difference was analyzed by one-way ANOVA. *p < 0.05, **p < 0.01, ***p < 0.001. *λ*_ex_ = 450 nm, *λ*_em_ = 630–710 nm. Scale bars: 40 μm. In (c)–(f) and (h)–(k), the error bars indicate the SD.

Finally, PC12 cells were used to construct an oxygen glucose deprivation/re-oxygenation (OGD/R) model and simulate cells undergoing ischemia-reperfusion,^49^ with which the relationship between cellular OGD/R and ferroptosis were explored (Fig. 5a). According to Fig. 5b and c, the red channel fluorescence intensity in the OGD/R group decreases significantly compared to the control group (*p* < 0.001). However, the fluorescence intensity of the group with Fer-1 is again significantly higher compared to the group without Fer-1 (*p* < 0.001). Therefore, the cellular ischemia-reperfusion process was accompanied by ferroptosis. It should be noted that the red channel fluorescence intensity of the control group with the addition of Fer-1 is slightly increased compared to the group without Fer-1, indicating the presence of ferroptosis among normal cells. The trends of Fe^2+^, MDA, and GSH levels are comparable with those observed for the fluorescence intensity (Fig. 5d–f), indicating the capacity of the probe to monitor ferroptosis progression induced by OGD/R. The p62–Keap1–Nrf2 signaling pathway was examined to further explore the pathological mechanisms by which the cellular ischemia-reperfusion process induces ferroptosis.^47,50^ Western blotting assay shown in Fig. 5g–k indicated that the p62–Keap1–Nrf2 signaling pathway was activated in the OGD/R cells, while GPX4 expression was significantly downregulated. After incubation with Fer-1, the p62–Keap1–Nrf2 signaling pathway was suppressed and GPX4 was upregulated. The p62–Keap1–Nrf2 signaling pathway and the GPX4 expression in the control group indicated no significant changes with or without the addition of Fer-1. Therefore, the ischemia-reperfusion process inhibited GPX4 and in turn affected the p62–Keap1–Nrf2 signaling pathway to induce ferroptosis.

### *In vivo* imaging of cerebral apoplexy in mice

**Fig. 6:**
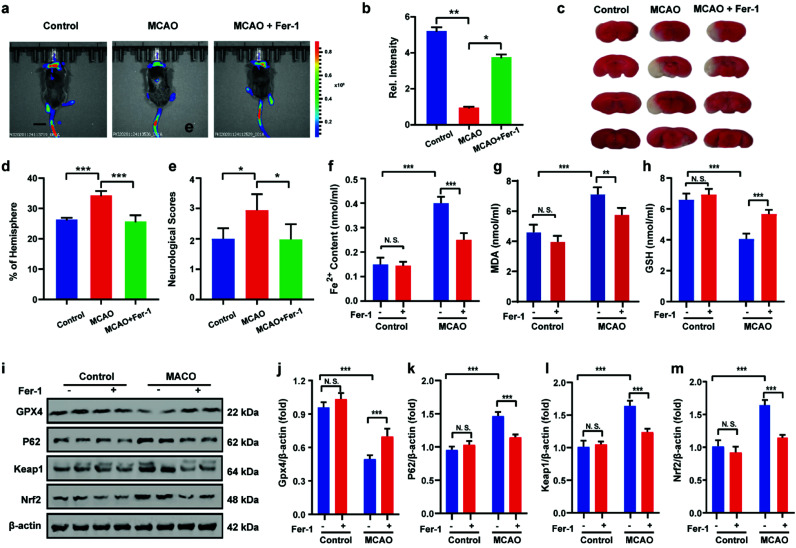
Visual imaging MCAO in living mice model. (a) Mice using MCAO models with different treatments: control group (mice not undergoing MCAO); MCAO group (mice undergoing MCAO); Fer-1 group (injection of Fer-1 to mice tail veins). Scale bar = 2 cm. (b) Histograms of average fluorescent intensities in (a); (c) infarct size in the ipsilateral hemisphere was measured 3 days after stroke onset using TTC staining, then normalized to the contralateral hemisphere and expressed as a percentage. (d) Bar graphs represent the statistic results. (e) Neurological score test; the content of (f) Fe^2+^, (g) MDA and (h) GSH in living mice MCAO model under different conditions in (a). (i–m) Western blotting analysis of GPX4, p62, Keap1, and Nrf2 in living mice MCAO model under different conditions: control group (mice not undergoing MCAO); MCAO group (mice undergoing MCAO); Fer-1 group (injection of Fer-1 to mice tail veins). In (b), (d)–(h) and (j)–(m), the error bars indicate the SD. Difference was analyzed by one-way ANOVA. *p < 0.05, **p < 0.01, ***p < 0.001. *λ*_ex_ = 450 nm, *λ*_em_ = 630–710 nm.

After uncovering the relationship between ischemia-reperfusion and ferroptosis, the cerebral apoplexy in mice was simulated using a middle cerebral artery occlusion (MCAO) model. Prior to imaging, the biocompatibility of HL-H_2_S in each tissue was assessed. After intravenous injection of HL-H_2_S, the different organs, including brain, heart, liver, spleen, lung, and kidney were stained by hematoxylin and eosin (H&E). As shown in Fig. S13,†HL-H_2_S exerted no obvious organ or tissue damage, indicating its good tissue biocompatibility for *in vivo* imaging analysis in mice. The findings acquired from the mouse MCAO model were like those obtained from the cellular model. The fluorescence intensity in the brains of mice in the MCAO model group decreased significantly compared to that of normal mice (*p* < 0.01). However, adding ferroptosis inhibitor Fer-1 caused a significant rebound of fluorescence intensity in the brains of mice (Fig. 6a and b, *p* < 0.05), indicating that MCAO in mice could lead to ferroptosis in their brains. 2,3,5-Triphenyltetrazolium chloride (TTC, measures tissue viability used to evaluate infarct size) staining and behavioral experiment results are in high agreement with *in vivo* imaging, indicating the viability of the mouse MCAO model (Fig. 6c–e). The changes in Fe^2+^, MDA, and GSH levels again demonstrate that ferroptosis occurred in the brain of mice during stroke (Fig. 6f–h). Similarly, the relationship between ferroptosis and GPX4 activity and the p62–Keap1–Nrf2 signaling pathway was explored in mice, and the results were consistent with those at the cellular level. Therefore, the MCAO process inhibits GPX4 activity in mice, which in turn affects the p62–Keap1–Nrf2 signaling pathway and ultimately leads to ferroptosis (Fig. 6i–m).

## Conclusions

In summary, a H_2_S triggered and H_2_S releasing NIR fluorescent probe HL-H_2_S was developed, which exhibits dual response to viscosity and H_2_S. After reacting with H_2_S, the probe releases COS through 1,6-elimination, which in turn releases H_2_S catalyzed by CA. *In vitro* experiments confirm a low detection limit of 1.3 nM, significant fluorescence enhancement and good selectivity amongst various ROS/RNS species. In addition, the probe exhibited excellent characteristics in terms of large Stokes shift (216 nm), favourable water solubility, excellent pH stability, and low cytotoxicity. While the cell experiments indicated that the progression of erastin induced ferroptosis in cells with and without HL-H_2_S were not significantly different. In contrast, the progression of ferroptosis was significantly promoted when the H_2_S release from HL-H_2_S was inhibited using AZ. Significantly, by using H_2_S triggered and H_2_S releasing mechanisms during the imaging process, the probe could avoid the induction of ferroptosis. Therefore, the probe is capable of *in situ* high-fidelity ferroptosis analysis, making it a reliable tool for the comprehensive and accurate understanding of ferroptosis progression. A cellular OGD/R model was constructed, and a mouse MCAO model was built to simulate MCAO in mice, and ferroptosis inducer erastin and ferroptosis inhibitor Fer-1 were used to regulate the progression of ferroptosis during MCAO. Confocal imaging revealed that the MCAO process could induce ferroptosis.

## Data availability

All data supporting this study are provided as ESI† accompanying this paper.

## Author contributions

Tianyu Liang: methodology, investigation, data curation, validation, formal analysis, writing – original draft. Taotao Qiang: project administration, writing – review & editing. Longfang Ren: data curation, investigation. Fei Cheng: validation. Baoshuai Wang: data curation, formal analysis. Mingli Li: software, data curation. Wei Hu: investigation, data curation, formal analysis, writing – original draft. Tony D. James: conceptualization, writing – review & editing, supervision.

## Conflicts of interest

TDJ acts as an academic consultant for TQ as part of a guest professorship at SUST.

## Supplementary Material

SC-013-D1SC05930K-s001
