# Supplementary material for: Near-infrared fluorescent probe for hydrogen sulfide: high-fidelity ferroptosis evaluation in vivo during stroke
Source: Chem Sci. 2022 Feb 21;13(10):2992–3001. doi: 10.1039/d1sc05930k (PMC8905919; doi:10.1039/d1sc05930k)
Supplement: SC-013-D1SC05930K-s001 [file SC-013-D1SC05930K-s001.pdf]

Electronic Supplementary Material (ESI) for Chemical Science.
This journal is © The Royal Society of Chemistry 2022

Near-infrared Fluorescent Probe for Hydrogen Sulfide: High-Fidelity Ferroptosis Evaluation *In Vivo* During Stroke

Tianyu Liang,[a]‡ Taotao Qiang,[a,]*‡ Longfang Ren,[a] Fei Cheng,[a] Baoshuai Wang,[a] Mingli Li,[a] Wei Hu[a,b,]* and Tony D. James[b,c,]*

[a] *College of Bioresources and Materials Engineering, Shaanxi Collaborative Innovation Center of Industrial Auxiliary Chemistry & Technology, Shaanxi University of Science & Technology, Xi'an, 710021, China.*

[b] *Department of Chemistry, University of Bath, Bath, BA27AY, United Kingdoms.*

[c] *School of Chemistry and Chemical Engineering, Henan Normal University, Xinxiang 453007, China.*

‡ These authors contributed equally.

*Corresponding author.

E-mail: t.d.james@bath.ac.uk (Tony D. James), qiangtt515@163.com (Taotao Qiang) and huwchem@163.com. (W. Hu).

Table of Contents

1. General Information on Materials and Methods 3
Instruments and materials 3
Spectroscopic measurements 3
Determination of the detection limit 3
Quantum yield measurements 4
Viscosity determination and fluorescence measurements 4
Methylene blue method (MB) assay of H_2S 4
Cytotoxicity assay 5
Cell culture and imaging 5
Ferroptosis model 5
Measurement of the biomarkers of ferroptosis 5
Western blotting assay 6
Calculation of mean fluorescence intensity 6
OGD/R model 6
Middle cerebral artery occlusion (MCAO) model 7
Measurement of infarct volume and neurological deficit 7
Histological staining of the tissue slices 7
In vivo imaging studies 8
Statistical analysis 8
2. Synthesis of Probe HL-H_2S 9
3. Supplementary Figures 13
4. Supplementary Table 26
5. References 27
6. ^{1}H NMR Spectra, ^{13}C NMR Spectra, and HRMS Spectra of Compounds 28

1. General Information on Materials and Methods

Instruments and materials

Unless otherwise stated, all solvents and reagents were purchased from commercial suppliers and were used as received without further purification. The reactions were performed in standard glassware. All aqueous solutions were prepared in ultrapure water with a resistivity of 18.25 MΩ·cm (purified by Milli-Q system, Millipore). Column chromatography was performed using silica gel 60 (230 ± 400 mesh, 0.040 ±0.063 mm) from Dynamic Adsorbents. NMR spectra were recorded on a Bruker-400 spectrometer, using TMS as an internal standard. High-resolution mass spectrometry was performed with LTQ FT Ultra (Thermo Fisher Scientific, America) in MALDI-DHB mode. Absorption spectra were recorded with a UV-vis spectrophotometer (Shimadzu UV-2550, Japan), and fluorescence spectra were obtained with a fluorimeter (Shimadzu RF-6000, Japan). Fluorescence imaging of mice was performed on an IVIS Lumina LT Series III small animal optical *in vivo* imaging system (U.S.A.) with an excitation filter of 500 nm and an emission filter of 650 nm. Experimental mice were anesthetized on an R500IE anesthesia machine. Living Image 4.5 software (PerkinElmer) was used for data analysis.

Spectroscopic measurements

Small amount of probe **HL-H_2S** was dissolved in DMSO to prepare the stock solutions (5.0×10^{-2} M). Unless otherwise mentioned, all the measurements for probe **HL-H_2S** target reaction were tested in PBS buffer (10 mM, pH 7.4, containing 2% DMSO and 80% glycerol). After adding NaHS and incubating at 37 °C for 40 min in a thermostat, a 500 μL aliquot of the reaction solution was transferred to a quartz cell with an optical length of 1 cm for the measurement of absorbance or fluorescence. The excitation wavelength was 450 nm. For the selectivity assay, superoxide anion ($O_2^{\bullet-}$), ·OH, $ONOO^-$, NO, H_2O_2, and NO_2^- were generated according to previous report.[1]

Determination of the detection limit

The limit of detection (LOD) for hydrogen sulfide was calculated based on the following equation:

$$LOD = 3\sigma/k$$

Where σ represents the standard deviation and k represents the slope of the titration spectra curve among the limited range.

Quantum yield measurements

The measurement of the fluorescence quantum yield was measured by using an ethanol solution of rhodamine B as a standard (10 μM, Φ_r = 0.71) and using the following equation[2].

$$\Phi_s = \frac{A_r \cdot F_S \cdot n_s^2}{A_s \cdot F_r \cdot n_r^2} \Phi_r \quad (A \leq 0.05)$$

Where s and r represent the sample to be tested and the reference dye, respectively. A represents the absorbance at the maximum absorption wavelength, F represents the fluorescence spectrum integral at the maximum absorption wavelength excitation, and n represents the refractive index of the sample to be tested or the reference dye solvent.

Viscosity determination and fluorescence measurements

The solvents were obtained by mixing a water-glycerol system in different proportions. Measurements were carried out with an NDJ-8S rotational viscometer, and each viscosity value was recorded. The relationship between the fluorescence emission intensity of the probe **HL-H_2S** and the viscosity of the solvent is well expressed by the Förster-Hoffmann equation as follows: $\log I_f = C + x\log \eta$, Where η is the value of viscosity, I is the emission intensity, C is a constant, and x represents the sensitivity of the probe **HL-H_2S** to viscosity[3].

Methylene blue method (MB) assay of H_2S

The methylene blue method was carried out as previously described. Briefly, the vials were evaluated in PBS buffer (10 mM, pH 7.4, containing 2% DMSO and 80% glycerol) containing CA (100 µg/mL) and AZ (50 µM). After adding probe **HL-H_2S** and NaHS and then incubating at 37 °C for 60 min in a thermostated bath equal volumes of MB solution were added. After reaction for 30 min, the absorbance of the mixture was determined at 670 nm. Methylene blue (MB) solution: 20% of Zn $(OAc)_2$ (1%, w/v), 40% of $FeCl_3$ (30 mM in 1.2 M HCl) and 40% of *N*, *N*-dimethyl-*p*-phenylenediamine dihydrochloride (20 mM in 7.2 M HCl). H_2S concentration in each sample was calculated against the H_2S calibration curve made by measuring a series of

known NaHS solutions.

Cytotoxicity assay

The cytotoxicity was evaluated by MTT assay. Briefly, PC12 cells (from Procell Life Science & Technology Co., Ltd.) were cultured in DMEM in 96-well microplates in incubator for 24 h. The medium was next replaced by fresh DMEM containing various concentrations of **HL-H_2S** (0-30 μM). Each concentration was tested in five replicates. Cells were rinsed twice with phosphate buffer saline (PBS) 24 h later and incubated with 0.5 mg/mL MTT reagent for 4 h at 37 °C. The absorbance at 490 nm was measured by microplate reader (Synergy 2. BioTek Instruments Inc.). Cell survival rate was calculated by $A/A_0 \times 100$ % (A and A_0 are the absorbance of the **HL-H_2S** labelled group and the control group, respectively).

Cell culture and imaging

PC12 cells were cultured with DMEM supplemented with 10% (v/v) fetal bovine serum (Gibco), 100 U/mL penicillin, and 100 μg/mL streptomycin in a humidified atmosphere with 5/95 (v/v) of CO_2/air at 37 °C. One day before imaging, cells were detached with a treatment of 0.2% (w/v) trypsin-EDTA solution (Gibco) and suspended in culture media. The cell suspension was then transferred to confocal dishes to grow with adherence. For imaging, PC12 cells at 80% confluence were harvested by scraping and transferred to confocal dishes to grow with adherence.

Ferroptosis model

Cells were treated with erastin (10 μM) for appropriate time to induce ferroptosis. After that the culture media were removed, and the cells were washed with serum-free media and then incubated with **HL-H_2S** (10 μM) for different treated. Imaging was performed with confocal microscope.

Measurement of the biomarkers of ferroptosis

Cellular Fe^{2+} level was measured by using an iron assay kit (Sigma-Aldrich) according to the manufacturer's instructions.[5] Malondialdehyde (MDA, Sigma-Aldrich) level was measured by using an MDA assay kit (Sigma-Aldrich) according to the manufacturer's instructions and glutathione peroxidase 4 (GPX4, Sigma-Aldrich) level was measured by using an GSH assay kit (Sigma-Aldrich) according to the

manufacturer's instructions[6]. The results were normalized to total protein concentrations.

Western blotting assay

Western blotting was carried out as previously described.[7] Cortical sections 1.0 to 2.0 mm from ipsilateral brain tissue was harvested and homogenized in cold RIPA buffer (C1053, Applygen, Beijing, China) plus protease inhibitor cocktail (G2006, Servicebio, Wuhan, China). The homogenates were centrifuged at 4 °C at 10, 000 × g for 30 min, and then the supernatants were harvested. Protein content was determined with the BCA kit (G2026, Servicebio, Wuhan, China). Protein samples (20 μl/lane) were separated by electrophoresis on 4– 15% sodium dodecyl sulfate-polyacrylamide gels and then transferred onto PVDF membranes (Millipore, Billerica, MA, USA). Membranes were then put into 5% non-fat milk with PBS/0.1% Tween and blocked for 1 h. The primary antibodies against GPX4 (Abcam, Cambridge, MA, USA), Keap1 (Abcam), Nrf2 (Abcam), p62 (Cell Signaling Technology, Boston, USA) at 4 °C. After washing with PBS/0.1% Tween, the membrane was incubated with IRDye-labeled secondary antibody (Li-Cor Bioscience, USA) at room temperature for 1–2 h. Images were acquired with the Odyssey Western Blot Analysis system (LI-COR, Lincoln, NE, USA). The relative band intensity was calculated using Quantity One v4.6.2 software (Bio-Rad Laboratories, Hercules, USA) and then normalized to the β-actin loading control. All above experiments were operated three times.

Calculation of mean fluorescence intensity

The mean fluorescence density was measured by Image-Pro Plus (v. 6.0) and calculated via the equation (mean density = $IOD_{sum}/area_{sum}$), where IOD and area were integral optical density and area of the fluorescent region.

OGD/R model

OGD/R model of cells was performed by oxygen and glucose deprivation/reperfusion. PC12 cells at 80% confluence were harvested by scraping and transferred to confocal dishes to grow with adherence. When the cells are adherent, the culture medium is changed to sugar-free DMEM and cultured in a three-gas incubator for 5 h without oxygen. Afterwards, these cells were incubated with high-glucose DMEM in a 5 % CO_2 and 95% O_2 atmosphere for 5 h. Then, the cells were incubated

with **HL-H_2S** (10 μM) for 30 minutes. Wash cells three times with PBS for confocal imaging.

Middle cerebral artery occlusion (MCAO) model

MCAO was induced using a previously described method with slight modifications.[8] In brief, C57BL/6J wild-type mice were anesthetized with 5% isoflurane in O_2 by facemask, followed by ligation of the left middle cerebral artery with 6-0 monofilament (Doccol Corp., Redlands, CA, USA). After 1 h of occlusion, the monofilament was removed to initiate reperfusion. A homeothermic heating pad was employed to monitor and stabilize the mice body temperature at 37 ± 0.5 °C. The same procedure, but without monofilament ligation, was performed on sham-operated mice.

Measurement of infarct volume and neurological deficit

Mice were deeply anesthetized and euthanized with an overdose of isoflurane and decapitated MCAO. The brains were collected after transcranial perfusion by saline followed with 4% paraformaldehyde. Brain tissues were cut into 1-mm coronal sections, and then dipped in 2% 2,3,5-triphenyltetrazolium chloride (TTC) (17779, Sigma-Aldrich, United States) for staining. The infarct volume was measured and analyzed by a blinded observer using ImageJ v1.37 (NIH, Bethesda, MA, United States), as described previously,[9] then was normalized and presented as a percentage of the non- ischemic hemisphere to correct for edema.[9] Neurological deficit scores were evaluated MCAO as described previously.[10] The score ranged from 0 (without observable neurological deficit) to 4 (no spontaneous motor activity and loss of consciousness).

Histological staining of the tissue slices

After imaging, the mice were killed, and the brains and other tissues (brain, heart, liver, spleen, lung and kidney) were collected for tissue analysis. Through a series of standard procedures, including fixation in 10% neutral buffered formalin, embedding into paraffin and sectioning at 3 μm thickness, the tissues were stained with hematoxylin-eosin (H&E). Thereafter, the prepared slices were examined by a digital microscope.

In vivo imaging studies

All animal procedures were performed in accordance with the Guidelines for Care and Use of Laboratory Animals of South-Central University of Nationalities and experiments were approved by the Animal Ethics Committee of College of Biology (South-Central University of Nationalities). Wild-type C57BL/6J mice (n = 300; 25–30 g) were purchased from Hubei Experimental Animal Research Center. (Hubei, China; No. 43004700018817, 43004700020932). All animal experimental protocols were approved by the Animal Experimentation Ethics Committee of South-Central University of Nationalities (No. 2020-scuec-043) and were conducted according to the Animal Care and Use Committee guidelines of South-Central University of Nationalities. Animals were housed in a room with controlled humidity (65 ± 5%) and temperature (25 ± 1 °C), under a 12/12-hour light/dark cycle with free access to food and water for at least 1 week before the experiments. After the model was successfully established, **HL-H_2S** (100 μL, 200 μM) was injected through the tail vein, and the mice were anesthetized with isoflurane before fluorescence imaging using a Bruker *in vivo* imaging system. Whereafter, the mice were anesthetized and dissected to remove the mouse brain tissue, and a 300 μm section was prepared with a microtome.

Statistical analysis

Statistical Product and Service Solutions (SPSS) software 19.0 was used for the statistical analysis. The error bars shown in the figures represented the mean ± s.d. Differences were determined with a one-way analysis of variance (ANOVA) followed by LSD test. Statistical significance was assigned at $*P < 0.05$, $**P < 0.01$ and $***P < 0.001$. Sample size was chosen empirically based on our previous experiences and pre-test results. No statistical method was used to predetermine sample size and no data were excluded. The numbers of animals or samples in every group were described in the corresponding figure legends. The distributions of the data were normal. All experiments were done with at least three biological replicates. Experimental groups were balanced in terms of animal age, sex, and weight. Animals were all caged together and treated in the same way. Appropriate tests were chosen according to the data distribution. Variance was comparable between groups in experiments described throughout the manuscript.

2. Synthesis of Probe HL-H_2S

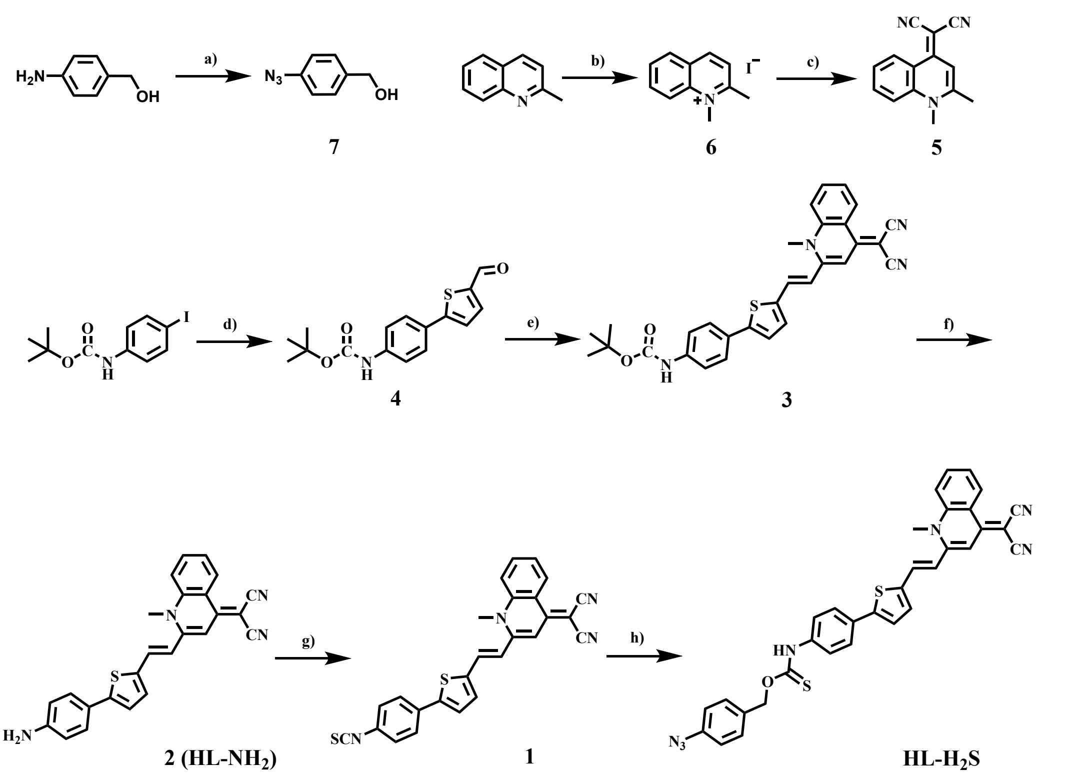

Scheme S1 Synthetic route of **HL-H_2S**. Reagents and conditions: a) NaN_3, $NaNO_2$, HCl, H_2O, 0 °C, 4 h; b) CH_3I, CH_3CN, reflux, 12 h; c) Malononitrile, C_2H_5ONa, C_2H_5OH, 0 °C→ r.t., 7 h; d) 5-formyl-2-thiopheneboronic acid, K_2CO_3, tetrakis (triphenylphosphine) palladium, toluene, CH_3OH, reflux, 8 h; e) Compound **5**, piperidine, C_2H_5OH, reflux, 24 h; f) CF_3COOH, DCM, 35 °C, 12 h; g) TCDI, NEt_3, THF, r.t., 24 h; h) Compound **7**, NaH, THF, 0 °C to r.t., 4 h.

Synthesis of compound **7**: was synthesized according to the previously reported route with some modifications,[11] A solution of $NaNO_2$ (1.4 g, 20.3 mmol) in 8 mL of water was added dropwise to a solution of 4-aminobenzyl alcohol (2.1 g, 17.5 mmol) in 4 M HCl (10 mL) at 0 °C. After stirring the mixture at this temperature for 1 h, a solution of NaN_3 (1.8 g, 28.9 mmol) in water (6 mL) was added slowly to the mixture at the same temperature. Stirring was continued for 1 hours below 5 °C and then at room temperature for another 3 hours. After the reaction, the mixture was extracted by dichloromethane and washed with water for three times. The organic phase was dried over anhydrous Na_2SO_4 and evaporated under reduced pressure. The crude residue was purified by column chromatography (1:30 v/v MeOH/dichloromethane) to obtain product (1.9 g), yield: 75%. ^1H NMR (400 MHz, $CDCl_3$, ppm) δ = 7.35 (d, J = 8.4 Hz,

2H), 7.02 (d, J = 8.4 Hz, 2H), 4.66 (s, 2H).

Synthesis of compound **5**: was synthesized according to the previously reported route with some modifications,[12] 2-methylquinoline (17.0 g, 118.7 mmol) and iodomethane (67.4 g, 474.9 mmol) were dissolved in absolute acetonitrile (125 mL). The reaction was reflux for 12 h. After the reaction, the solution was cooled to room temperature. The precipitate was filtered and collected in desiccators to give the product as yellow powder compound **6**. Malononitrile (6.1 g, 92.6 mmol) and compound **6** (12 g, 42.0 mmol) were dissolved in anhydrous ethanol (70 mL) at 0 °C and then a solution of sodium ethoxide (5.7 g, 84.2 mmol) in ethanol solution (40 mL) was added slowly to the mixture at this temperature. Stirring was continued for 2 hours below 5 ^{o}C and then at room temperature for another 5 hours. After the reaction, the solution was poured into ice water, the pH was adjusted to 7.4 by 2 mmol HCl. The crude residue was purified by column chromatography (1:40 v/v MeOH/dichloromethane) to give the product as dark yellow solid. Yield: 6.0 g (64.0%). ^{1}H NMR (400 MHz, DMSO-d_6, ppm) δ = 8.86-8.80 (m, 1H), 7.99 (d, J = 8.4 Hz, 1H), 7.88-7.84 (m, 1H), 7.58-7.52 (m, 1H), 6.75 (s, 1H), 3.87 (s, 3H), 2.63 (s, 3H).

Synthesis of compound **4**: N-Boc-4-iodoaniline (5.0 g, 15.7 mmol), 5-formyl-2-thiopheneboronic acid (3.4 g, 21.9 mmol), K_2CO_3 (10.8 g, 78.3 mmol), tetrakis (triphenylphosphine) palladium (1.8 g, 1.6 mmol) were dissolved in a mixture solvent (toluene/ methanol, 1:1 v/v, 70 mL). The mixture was refluxed for 8 h under an inert N_2 environment. After the reaction, solvent was removed and the crude residue was purified by column chromatography (1:1 v/v dichloromethane /petroleum ether) to give the product as yellow solid. Yield: 2.9 g (61.1%). ^{1}H NMR (400 MHz, $CDCl_3$, ppm) δ = 9.86 (s, 1H), 7.71 (d, J = 3.8 Hz, 1H), 7.59 (d, J = 8.4 Hz, 2H), 7.44 (d, J = 8.4 Hz, 2H), 7.32 (d, J = 3.8 Hz, 1H), 6.69 (s, 1H), 1.53 (s, 9H); ^{13}C NMR (100 MHz, $CDCl_3$, ppm) δ = 182.71, 154.25, 152.43, 141.74, 139.72, 137.63, 127.68, 127.20, 123.34, 118.70, 81.06, 28.32.

Synthesis of compound **3**: To compound **4** (1.5 g, 4.9 mmol) and compound **5** (1.7 g, 7.2 mmol) was added piperidine (1.0 mL, 9.9 mmol). The reaction mixture was then dissolved in ethanol (50 mL) and refluxed for 24 h. After the reaction, the solution was cooled to room temperature. A red precipitate appeared. The precipitate was filtered and the crude residue was purified by column chromatography (1:40 v/v MeOH/dichloromethane) to give the product as red solid. Yield: 1.5 g (60.0%). ^{1}H

NMR (400 MHz, DMSO-d_6, ppm) δ = 9.58 (s, 1H), 8.89 (d, J = 8.4 Hz, 1H), 8.03 (d, J = 8.4 Hz, 1H), 7.94-7.91 (m, 1H), 7.71 – 7.40 (m, 8H), 7.19-7.17 (m, 1H), 7.01 (s, 1H), 3.98 (s, 3H), 1.49 (s, 9H); ^{13}C NMR (100 MHz, DMSO-d_6, ppm) δ = 152.58, 152.06, 149.31, 146.17, 139.92, 139.19, 138.41, 138.35, 133.49, 132.77, 132.71, 132.37, 132.36, 126.81, 126.01, 124.95, 124.78, 120.35, 119.01, 118.38, 106.25, 79.33, 46.65, 37.47, 28.06; HR-MS (MALDI-DHB) calcd for $C_{30}H_{26}N_4O_2S$ $[M+Na]^+$: 529.16687, found: 529.16669.

Synthesis of compound **2**: Compound **3** (1.0 g, 2.0 mmol) was dissolved in absolute dichloromethane (100 mL) and then trifluoroacetic acid (8.0 mL) was added slowly. The reaction was stirred at 35 °C for 12 h. After the reaction, solvent was removed and the crude residue was purified by column chromatography (1:40 v/v MeOH/dichloromethane) to give the product as dark red solid. Yield: 0.6 g (74.8%). ^{1}H NMR (400 MHz, DMSO-d_6, ppm) δ = 8.88 (d, J = 8.4 Hz, 1H), 8.01 (d, J = 8.4 Hz, 1H), 7.92-7.89 (m, 1H), 7.64 – 7.55 (m, 2H), 7.49 (d, J = 3.6 Hz, 1H), 7.40 (d, J = 8.4 Hz, 2H), 7.28 (d, J = 3.6 Hz, 1H), 7.08-7.06 (m, 1H), 6.99 (s, 1H), 6.61 (d, J = 8.4 Hz, 2H), 5.55 (s, 2H), 3.96 (s, 3H); ^{13}C NMR (100 MHz, DMSO-d_6, ppm) δ = 152.33, 150.16, 149.94, 148.82, 139.67, 136.93, 133.92, 133.76, 133.22, 127.21, 125.39, 125.24, 121.92, 120.99, 120.84, 118.86, 118.10, 114.44, 106.52, 46.76, 37.92. HR-MS (MALDI-DHB) calcd for $C_{25}H_{18}N_4S$ $[M+Na]^+$: 429.11444, found: 429.11453.

Synthesis of compound **1**: Compound **2** (0.2 g, 0.49 mmol) and triethylamine (0.55 mL, 3.9 mmol) were dissolved in anhydrous THF (15 mL) under vigorous stirring, and then 1,1'-thiocarbonyldiimidazole (TCDI, 0.53 g, 2.9 mmol) in anhydrous THF (15 mL) was added dropwise to the aforementioned solution under nitrogen atmosphere. The reaction was allowed to stir for 24 h at room temperature and finally quenched with water. The mixture was extracted by dichloromethane and washed with water for three times. The organic phase was dried over anhydrous Na_2SO_4 and evaporated under reduced pressure. The crude residue was purified by column chromatography (1:50 v/v MeOH/dichloromethane) to generate the product as red solid. Yield: 0.11 g (50.0 %). ^{1}H NMR (400 MHz, DMSO-d_6, ppm) δ = 8.91 (d, J = 8.4 Hz, 1H), 8.05 (d, J = 8.4 Hz, 1H), 7.97 – 7.90 (m, 1H), 7.79 (d, J = 8.4 Hz, 2H), 7.72 – 7.59 (m, 4H), 7.52 (d, J = 8.4 Hz, 2H), 7.30-7.27 (m, 1H), 7.03 (s, 1H), 3.99 (s, 3H); ^{13}C NMR (100 MHz, DMSO-d_6, ppm) δ = 149.63, 147.39, 144.62, 143.93, 140.81, 139.71, 134.73, 134.07, 133.01, 132.96, 132.50, 130.06, 127.39, 127.20, 126.66, 126.50, 125.53, 125.32, 120.87,

120.73, 118.91, 108.04, 106.95, 47.45, 38.03. HR-MS (MALDI-DHB) calcd for $C_{26}H_{16}N_4S_2$ $[M+Na]^+$: 471.07086, found: 471.07040.

Synthesis of probe **HL-H$_2$S**: Compound **1** (70 mg, 0.16 mmol) and compound **7** (30 mg, 0.18 mmol) were dissolved in ice-cold anhydrous THF (10 mL), followed by the addition of sodium hydride (60% in mineral oil, 12 mg, 0.24 mmol). The mixture was stirred at 0 °C for 40 min, and then removed from ice bath, followed by stirring for another 3 h at room temperature. The solvent was evaporated under reduced pressure and the crude product was purified by column chromatography (1:50 v/v MeOH/dichloromethane), to generate the product as red solid. Yield: 30 mg (32.0%). ^{1}H NMR (400 MHz, DMSO-d_6, ppm) δ = 11.40 (s, 1H), 8.88 (d, J = 8.4 Hz, 1H), 8.02 (d, J = 8.4 Hz, 1H), 7.93-7.89 (m, 1H), 7.72 – 7.49 (m, 9H), 7.40 (s, 1H), 7.25 – 7.13 (m, 3H), 7.00 (s, 1H), 5.57-5.53 (m, 2H), 3.97 (s, 3H); ^{13}C NMR (100 MHz, DMSO-d_6, ppm) δ = 187.35, 152.56, 149.65, 149.61, 145.86, 139.90, 139.66, 133.99, 133.08, 132.81, 132.69, 130.73, 130.55, 130.11, 126.62, 126.31, 125.44, 125.28, 125.17, 121.07, 120.85, 119.94, 119.69, 119.64, 118.83, 106.83, 47.33, 37.97; HR-MS (MALDI-DHB) calcd for $C_{33}H_{23}N_7OS_2$ $[M+H]^+$: 598.14783, found: 598.14935.

3. Supplementary Figures

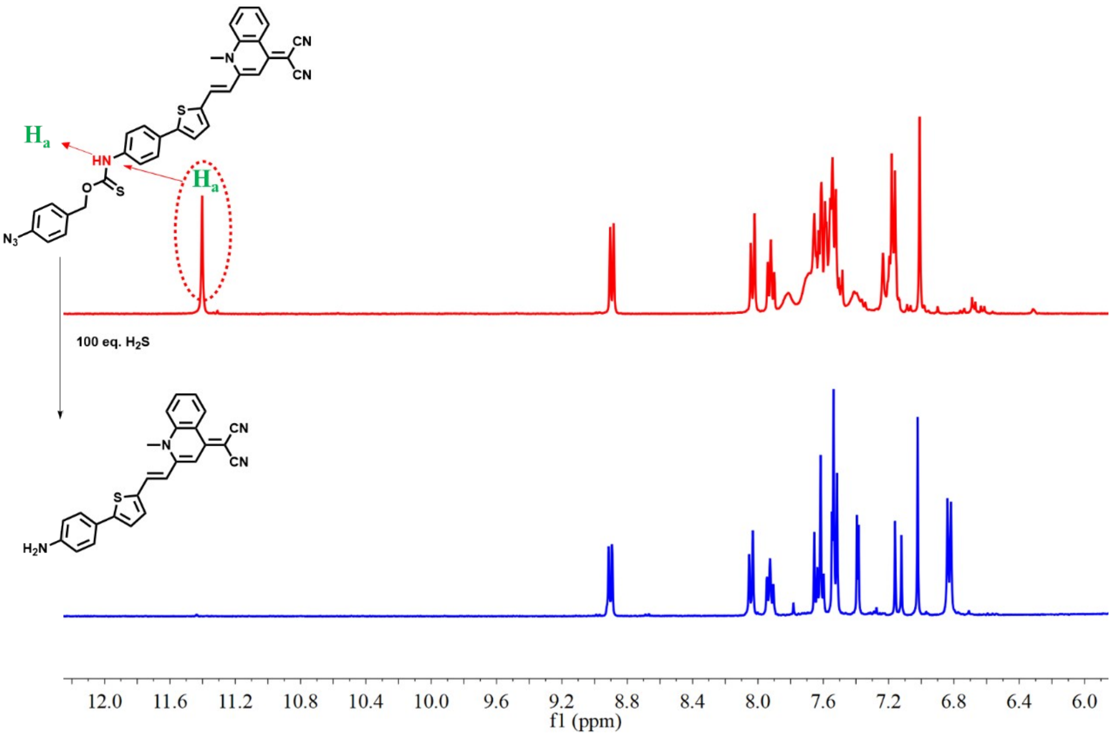

Fig. S1. The ^{1}H NMR spectra of probe **HL-H_2S** in the absent and present of 100 equiv NaHS.

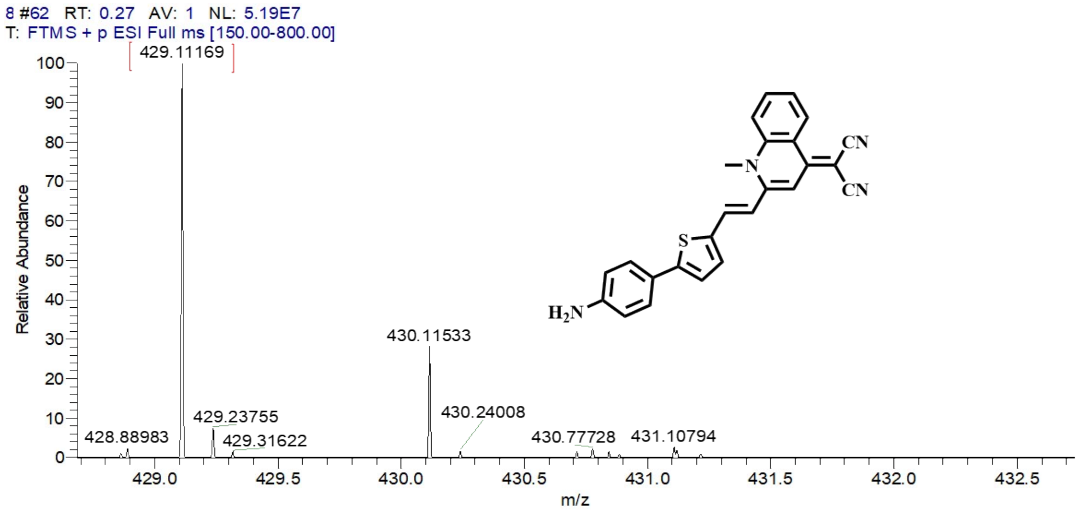

Fig. S2. The HR-MS of product obtained by reaction of **HL-H_2S** and NaHS.

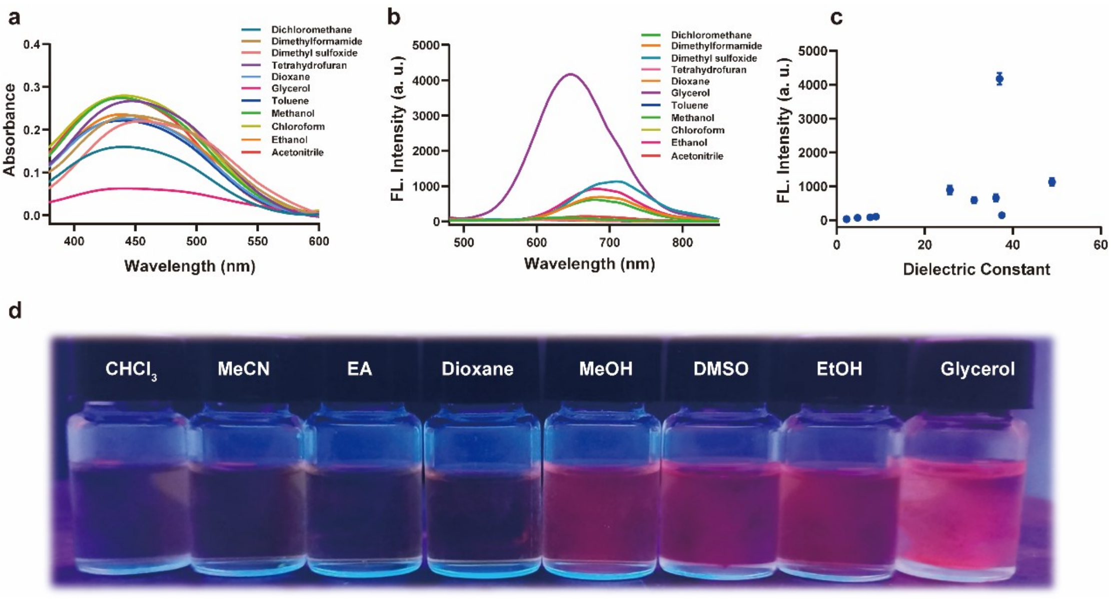

Fig. S3. UV-Vis absorption spectra (a) and fluorescence spectra (b) of **HL-NH$_2$** in different solvents. (c) Plot of the maximum emission peaks related to the dielectric constants of the medium. Solvents: dioxane, toluene, chloroform, tetrahydrofuran, dichloromethane, ethanol, methanol, dimethylformamide, glycerol, acetonitrile, dimethyl sulfoxide. (d) Photograph of **HL-NH$_2$** under 365 nm UV irradiation from a hand-held UV lamp in different solvents. Concentration of **HL-NH$_2$**: 10 μM. In c, data represent the mean of three replicates and the error bars indicate the SD.

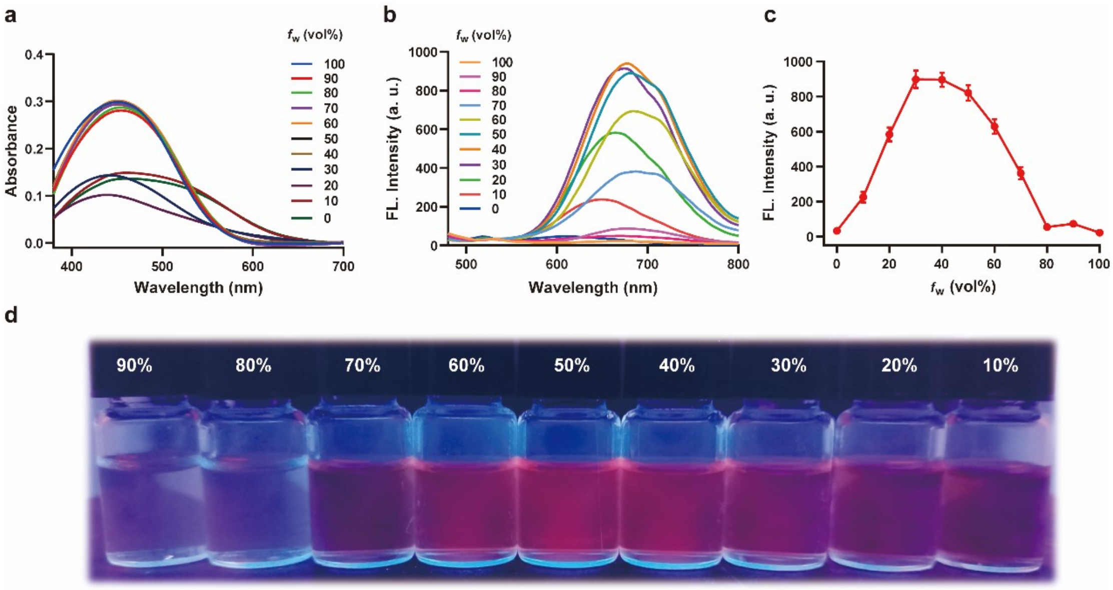

Fig. S4. UV-Vis absorption spectra (a) and fluorescence spectra (b) of **HL-NH$_2$** in a mixture of water-THF with different water fraction (f_w). (c) Variations in emission maximum of **HL-NH$_2$** with f_w, λ_{em} = 670 nm. (d) Photograph of **HL-NH$_2$** under 365 nm UV irradiation from a hand-held UV lamp with f_w. Concentration of **HL-NH$_2$**: 10 μM, λ_{ex} = 450 nm. In c, data represent the mean of three replicates and the error bars indicate the SD.

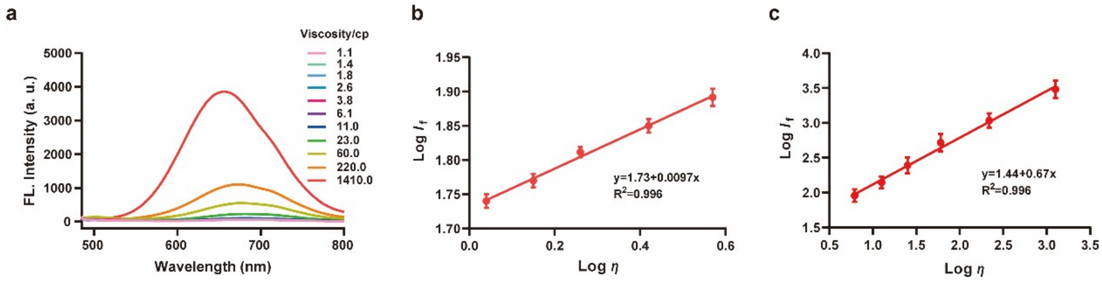

Fig. S5. (a) Solvent viscosity-dependent fluorescence changes of **HL-NH$_2$** in a water-glycerol system. The linear relationship between log I_f and log η of **HL-NH$_2$** in 0 ~ 40% (b) and 50 ~ 99% (c). (Fluorescence intensity I_f of a molecular rotor; viscosity η of the solvent; C is a concentration - and temperature - dependent constant), λ_{ex} = 450 nm, concentration of **HL-NH$_2$**: 10 μM. In b and c, data represent the mean of three replicates and the error bars indicate the SD.

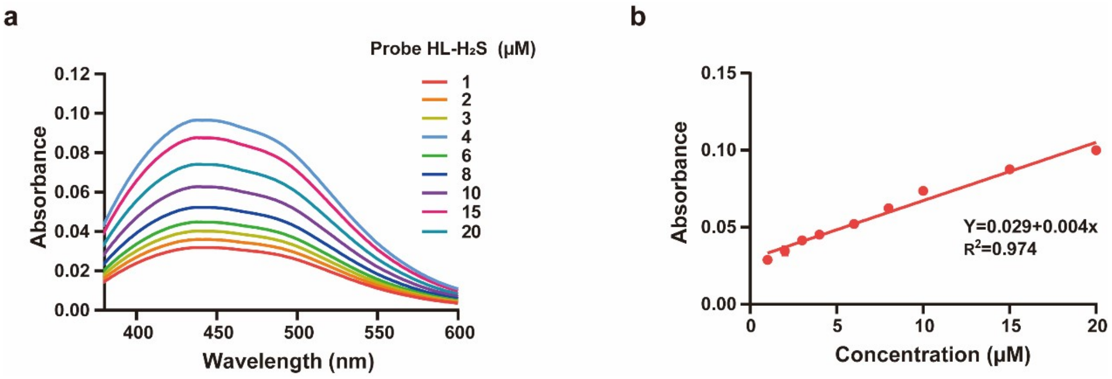

Fig. S6. (a) UV-Vis spectra of different concentration of probe **HL-H_2S** in PBS with 2% DMSO and 80% glycerol. (b) Linear relationship of the absorbance at 450 nm with the concentrations of **HL-H_2S** (1, 2, 3, 4, 6, 8, 10 ,15 and 20 μM). In b, data represent the mean of three replicates and the error bars indicate the SD.

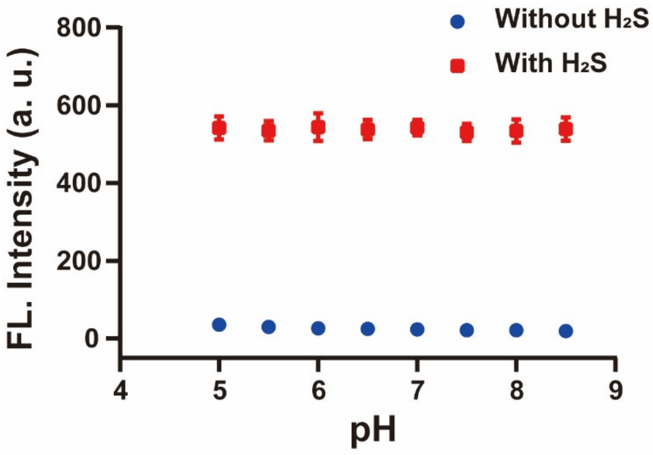

Fig. S7. Effects of pH on the fluorescence of **HL-H_2S** reacting with (red line) and without (green line) H_2S (100 μM). Data represent the mean of three replicates and the error bars indicate the SD.

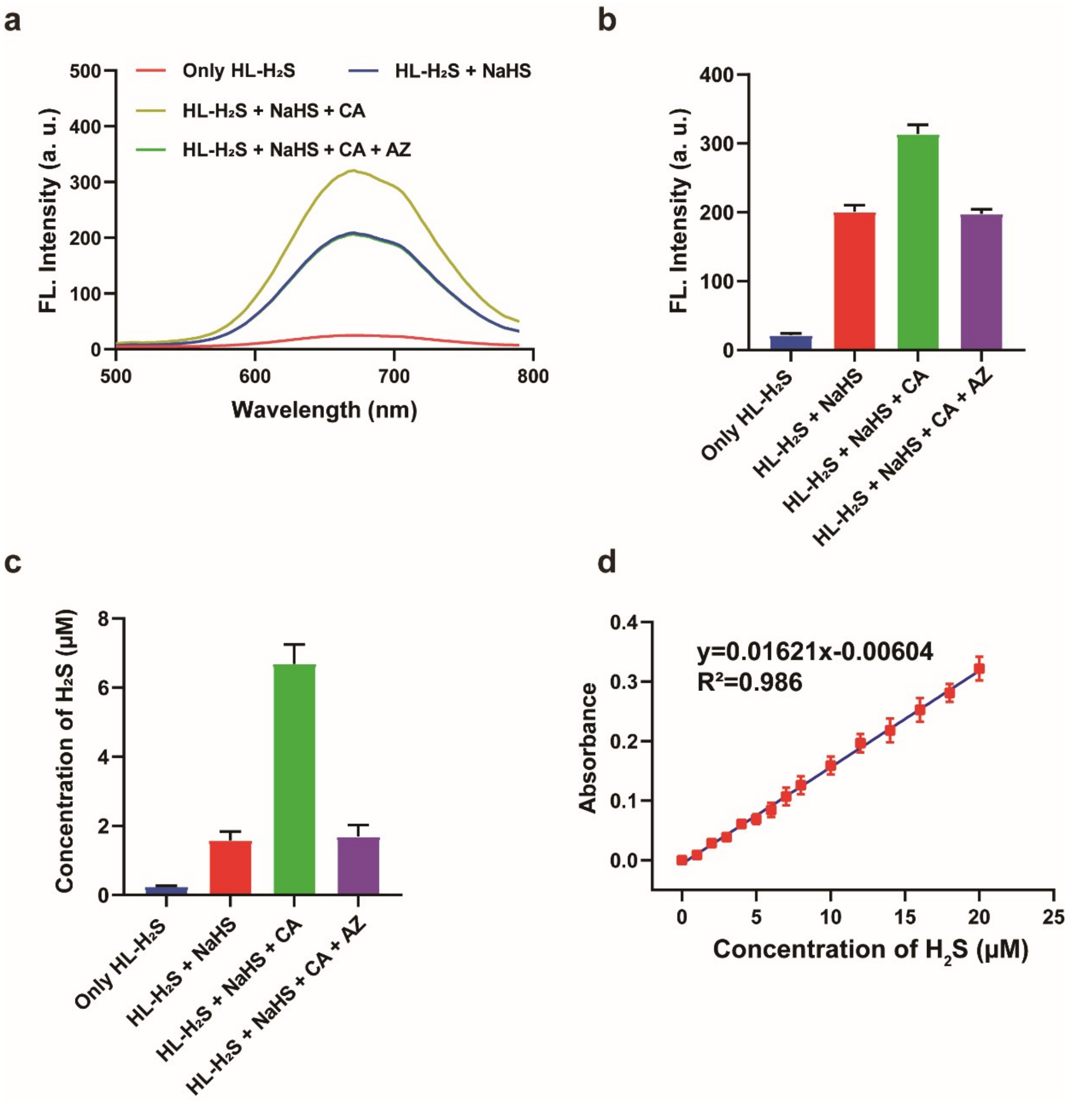

Fig. S8. The fluorescence spectra of probe **HL-H$_2$S** (10 μM) at varying conditions. Only **HL-H$_2$S**; **HL-H$_2$S** + NaHS (25 μM); **HL-H$_2$S** + NaHS (25 μM) + CA (100 μg/mL); **HL-H$_2$S** + NaHS (25 μM) + CA (100 μg/mL) + acetazolamide (AZ, 50 μM). (b) Histograms of average fluorescence intensity of (a). (c) The absorbance spectra of probe **HL-H$_2$S** (10 μM) at varying conditions. (d) Standard curve of NaHS at different concentrations (0 – 20 μM) was plotted and fitted. Test medium: PBS (pH = 7.4, containing 2% DMSO and 80% glycerol). λ_{ex} = 450 nm. Data represent the mean of three replicates and the error bars indicate the SD.

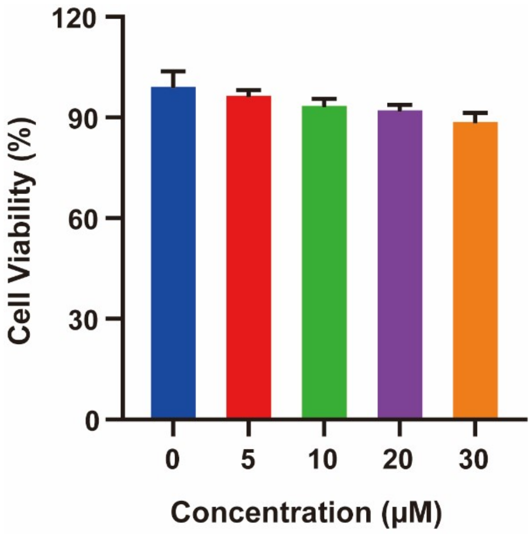

Fig. S9. MTT assay of PC12 cells treated with different concentrations of probe **HL-H_2S** (0, 5, 10, 20, 30 μM). Data represent the mean of three replicates and the error bars indicate the SD.

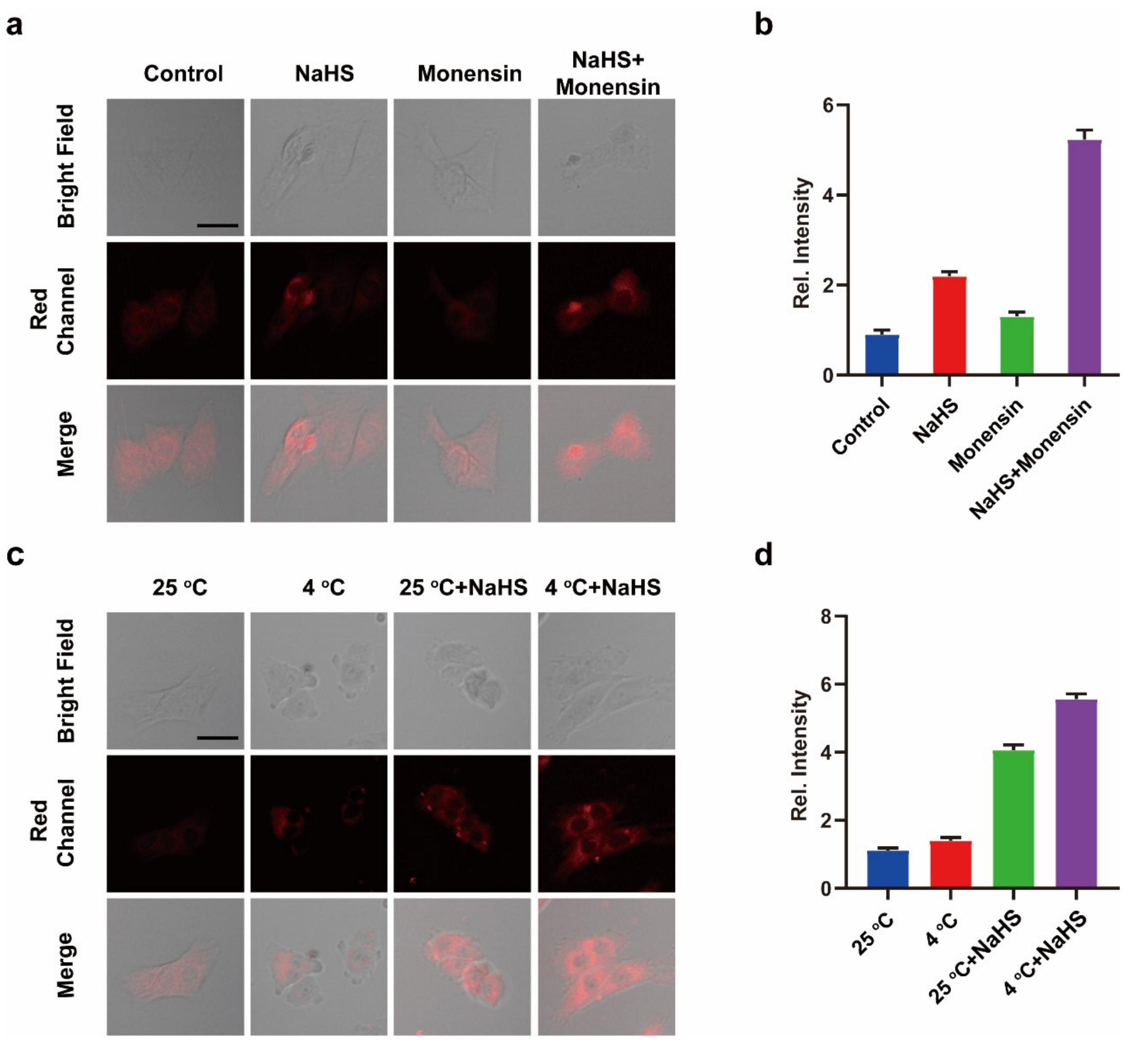

Fig. S10. (a) Confocal images of PC12 cells incubated with probe **HL-H_2S** (10 μM) in control group (only probe), NaHS (10 μM, H_2S donor), Monensin (10 μM, viscosity inducer), NaHS (10 μM) + Monensin (10 μM). (b) Histograms of average fluorescence intensity of (a). (c) Confocal images of PC12 cells incubated with probe **HL-H_2S** (10 μM) in 25 °C (lower viscosity), 4 °C (higher viscosity), 25 °C + NaHS (10 μM), 4 °C + NaHS (10 μM). (d) Histograms of average fluorescence intensity of (c). For the fluorescence change: data are presented as the mean ± SD (control: n = 40 cells from three cultures; NaHS: n = 37 cells from three cultures; Monensin: n = 33 cells from three cultures; NaHS + Monensin: n = 46 cells from three cultures; 25 °C: n = 43 cells from three cultures; 4 °C: n = 22 cells from three cultures; 25 °C + NaHS: n = 41 cells from three cultures; 4 °C + NaHS: n = 38 cells from three cultures). λ_{ex} = 450 nm, λ_{em} = 630-710 nm. Scale bars: 40 μm. In (b) and (d), data represent the mean of three replicates and the error bars indicate the SD.

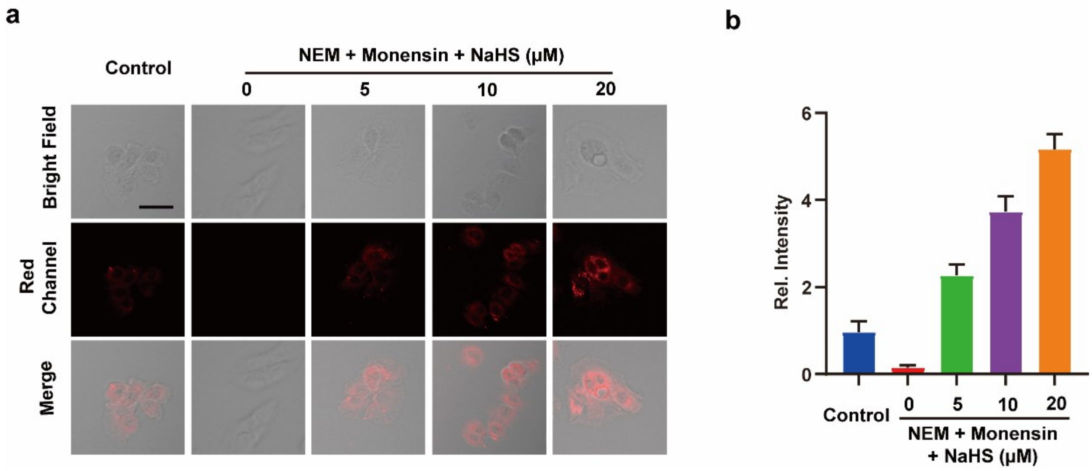

Fig. S11. (a) Confocal images of PC12 cells incubated with probe **HL-H_2S** (10 μM) in control group (only probe), **HL-H_2S** (10 μM) for 10 min and then, NEM (0.5 mM), monensin (10 μM), NaHS (0, 5, 10, 20 μM) for 40 min. (b) Histograms of average fluorescence intensity of (a). For the fluorescence change: control: n = 35 cells from three cultures; NEM (0.5 mM) + monensin (10 μM): n = 34 cells from three cultures; NEM (0.5 mM) + monensin (10 μM) + NaHS (5 μM): n = 33 cells from three cultures; NEM (0.5 mM) + monensin (10 μM) + NaHS (10 μM): n = 44 cells from three cultures; NEM (0.5 mM), monensin (10 μM), NaHS (20 μM): n = 46 cells from three cultures. λ_{ex} = 450 nm, λ_{em} = 630-710 nm. Scale bars: 40 μm. In b, data represent the mean of three replicates and the error bars indicate the SD.

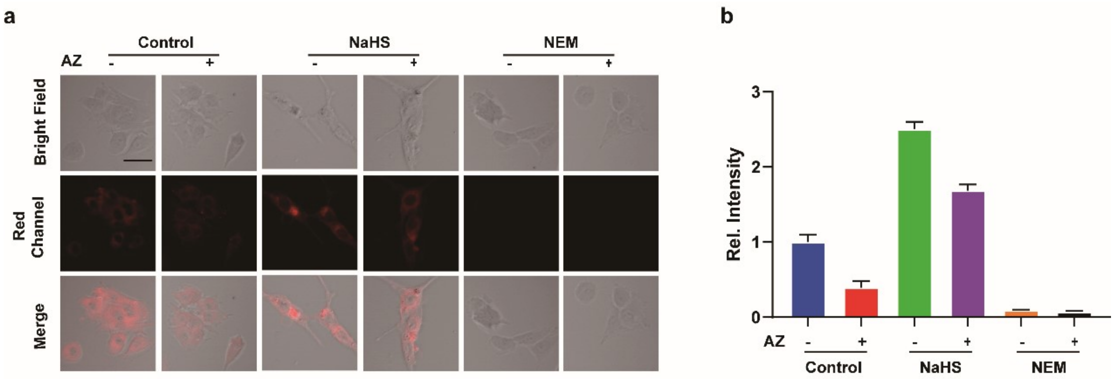

Fig. S12. (a) Confocal images of PC12 cells incubated with probe **HL-H$_2$S** (10 μM); AZ (50 μM) for 20 min and then **HL-H$_2$S** (10 μM) for 40 min; AZ (50 μM) for 20 min, and then NaHS (5 μM) for 20 min, finally **HL-H$_2$S** (10 μM) for 40 min; NaHS (5 μM) for 20 min and then **HL-H$_2$S** (10 μM) for 40 min; NEM (0.5 mM) for 30 min and then, **HL-H$_2$S** (10 μM); AZ (50 μM) and NEM (0.5 mM) for 30 min and then, **HL-H$_2$S** (10 μM) for 40 min; (b) Histograms of average fluorescence intensity of (a). For the fluorescence change: (untreated cells: n = 32 cells from three cultures; for AZ (50 μM) treated cells: n = 43 cells from three cultures; for NaHS (5 μM) treated cells: n = 42 cells from three cultures; for NaHS (5 μM) + AZ (50 μM) treated cells: n = 48 cells from three cultures; for NEM (0.5 mM) treated cells: n = 36 cells from three cultures; for NEM (0.5 mM) + AZ (50 μM) treated cells: n = 44 cells from three cultures). λ_{ex} = 450 nm, λem = 630-710 nm. Scale bars: 20 μm. In b, data represent the mean of three replicates and the error bars indicate the SD.

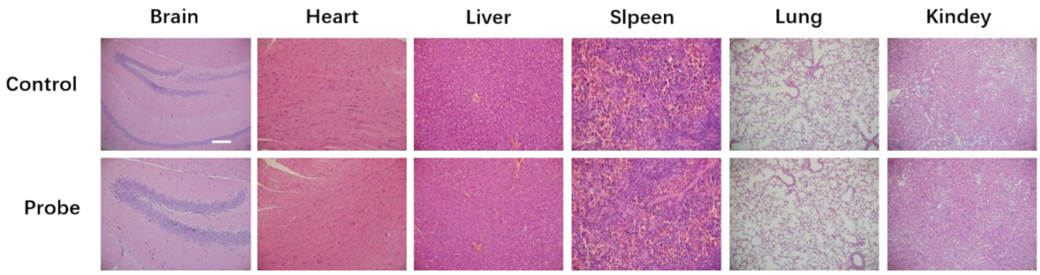

Fig. S13. H&E staining results of the main organs collected from the control group and probe **HL-H$_2$S** (100 μL, 200 μM) treated group. Scale bar: 50 mm.

4. Supplementary Table

Table S1. Photophysical properties of **HL-NH$_2$** in various solvents

Solvent	Dielectric Constant	λ_{max}^{abs} (nm)	ε ($M^{-1}cm^{-1}$)	λ_{max}^{em} (nm)	Φ
DCM	9.10	433	1.63*10^4	642	<0.01
DMF	36.71	432	2.37*10^4	683	0.18
DMSO	48.90	437	2.18*10^4	710	0.26
THF	7.58	434	2.71*10^4	642	<0.01
Dioxane	2.20	436	2.29*10^4	626	<0.01
Glycerol	37.00	430	0.64*10^4	646	0.67
Toluene	2.24	437	2.26*10^4	581	<0.01
CH_3OH	31.20	428	2.78*10^4	683	0.16
$CHCl_3$	4.90	433	2.85*10^4	613	<0.01
EtOH	25.70	430	2.43*10^4	687	0.23
CH_3CN	37.50	429	2.76*10^4	666	0.17

5. References

1. W. Hu, L. Zeng, S. Zhai, C. Li, W. Feng, Y. Feng and Z. Liu, *Biomaterials*, 2020, **241**, 119910.
2. Y. Chen, X. Shi, Z. Lu, X. Wang and Z. Wang, *Anal. Chem.*, 2017, **89**, 5278-5284.
3. L. Wang, Y. Xiao, W. Tian and L. Deng, *J. Am. Chem. Soc.*, 2013, **135**, 2903-2906.
4. Y. Hu, X. Li, Y. Fang, W. Shi, X. Li, W. Chen, M. Xian, H. Ma, *Chem. Sci.* **2019,** *10*, 7690-7694.
5. J. Cheng, T. Xu, C. Xun, H. Guo, R. Cao, S. Gao and W. Sheng, *Life Sci.*, 2021, **266**, 118905.
6. J. Li, K. Lu, F. Sun, S. Tan, X. Zhang, W. Sheng, W. Hao, M. Liu, W. Lv and W. Han, *J. Transl. Med.*, 2021, **19**, 96.
7. Q. Jiang, K. Wang, X. Zhang, B. Ouyang, H. Liu, Z. Pang and W. Yang, *Small*, 2020, **16**, 2001704.
8. X. X. Xiong, L. Xu, L. Wei, R. E. White, Y.-B. Ouyang and R. G. Giffard, *Stroke*, 2015, **46**, 2271-2276.
9. L. J. Gu, X. X. Xiong, H. F. Zhang, B. H. Xu, G. K. Steinberg and H. Zhao, *Stroke*, 2012, **43**, 1941-1946.
10. C. M. Stary, L. J. Xu, L. Li, X. Y. Sun, Y.-B. Ouyang, X. X. Xiong, J. Zhao and R. G. Giffard, *Mol. Cell. Neurosci.*, 2017, **82**, 118-125.
11. R.-Q. Han, Y.-B. Ouyang, L. J. Xu, R. Agrawal, A. J. Patterson and R. G. Giffard, *Anesth. Analg.*, 2009, **108**, 280-287.
12. T. Liang, P. Yang, T. Wu, M. Shi, X. Xu, T. Qiang and X. Sun, *Chin. Chem. Lett.*, 2020, **31**, 2975-2979.
13. M. Zhang, W. Du, X. Tian, R. Zhang, M. Zhao, H. Zhou, Y. Ding, L. Li, J. Wu and Y. Tian, *J. Mater. Chem. B.*, 2018, **6**, 4417-4421.

6. ^{1}H NMR Spectra, ^{13}C NMR Spectra, and HRMS Spectra of Compounds

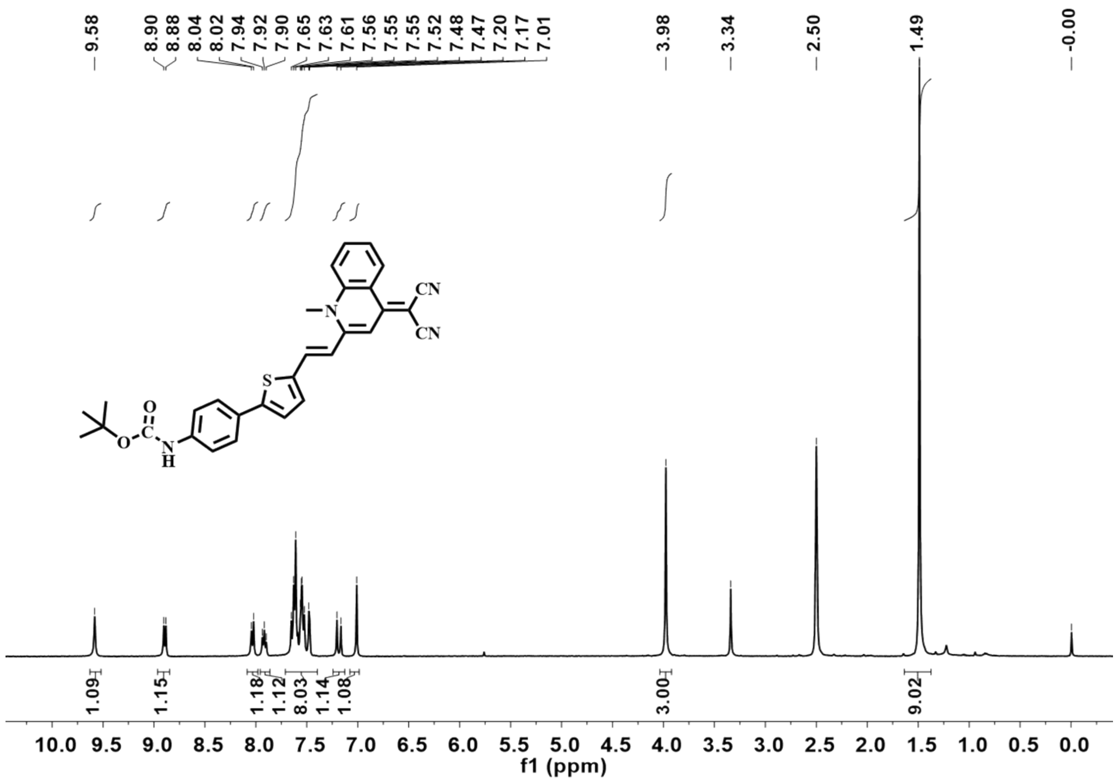

Fig. S14. ^{1}H NMR spectrum (400 MHz) of compound **3** in DMSO-d_6

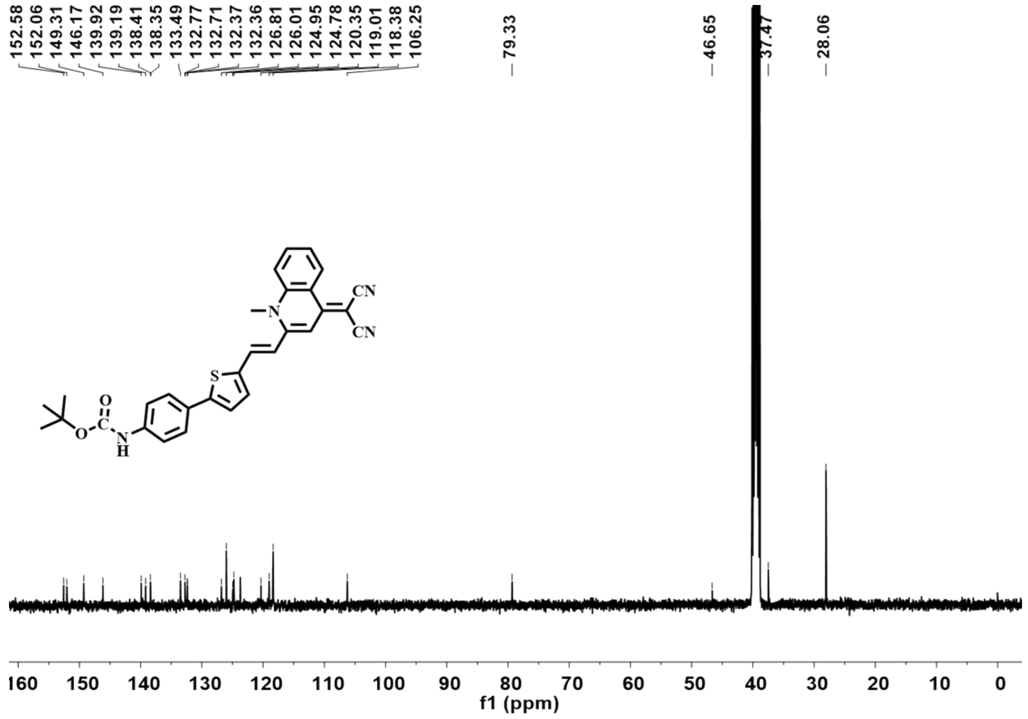

Fig. S15. ^{13}C NMR spectrum (100 MHz) of compound **3** in DMSO-d_6

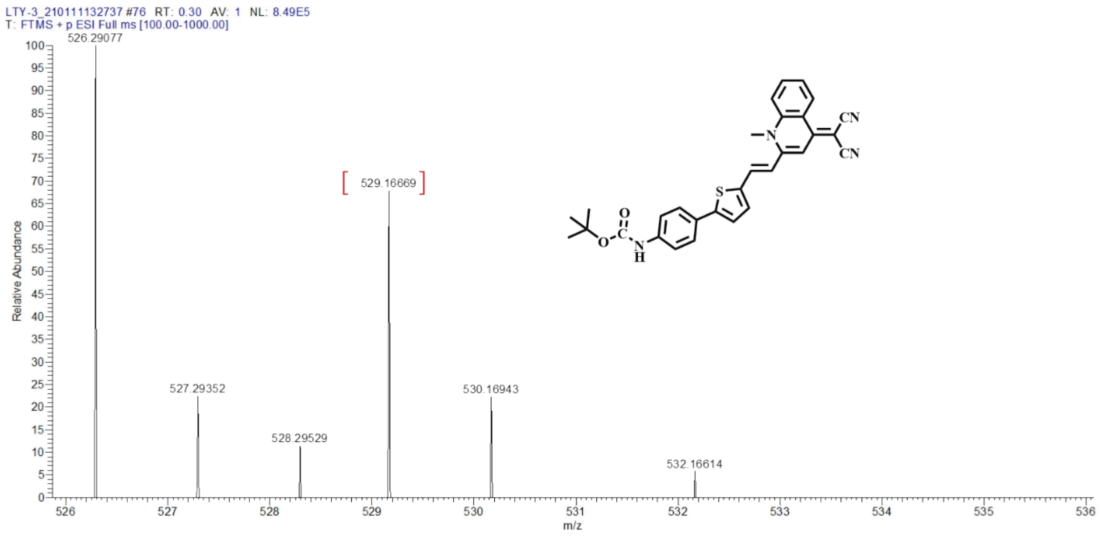

Fig. S16. HR-MS spectrum of compound **3**

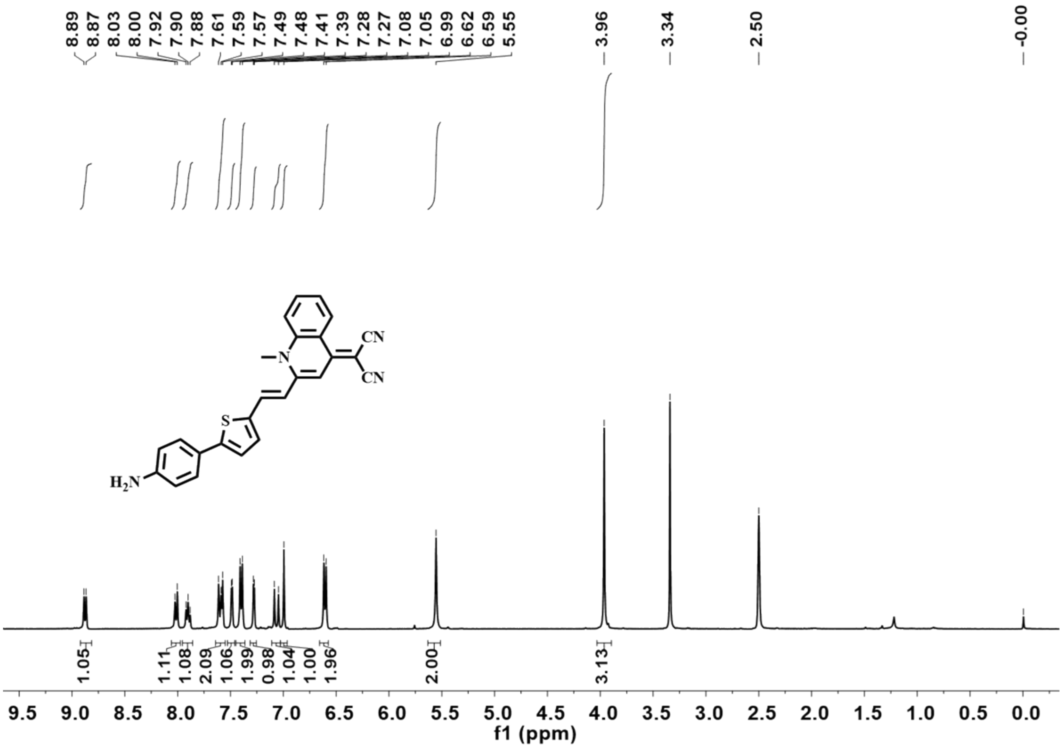

Fig. S17. ^{1}H NMR spectrum (400 MHz) of compound **2** in DMSO-d_6

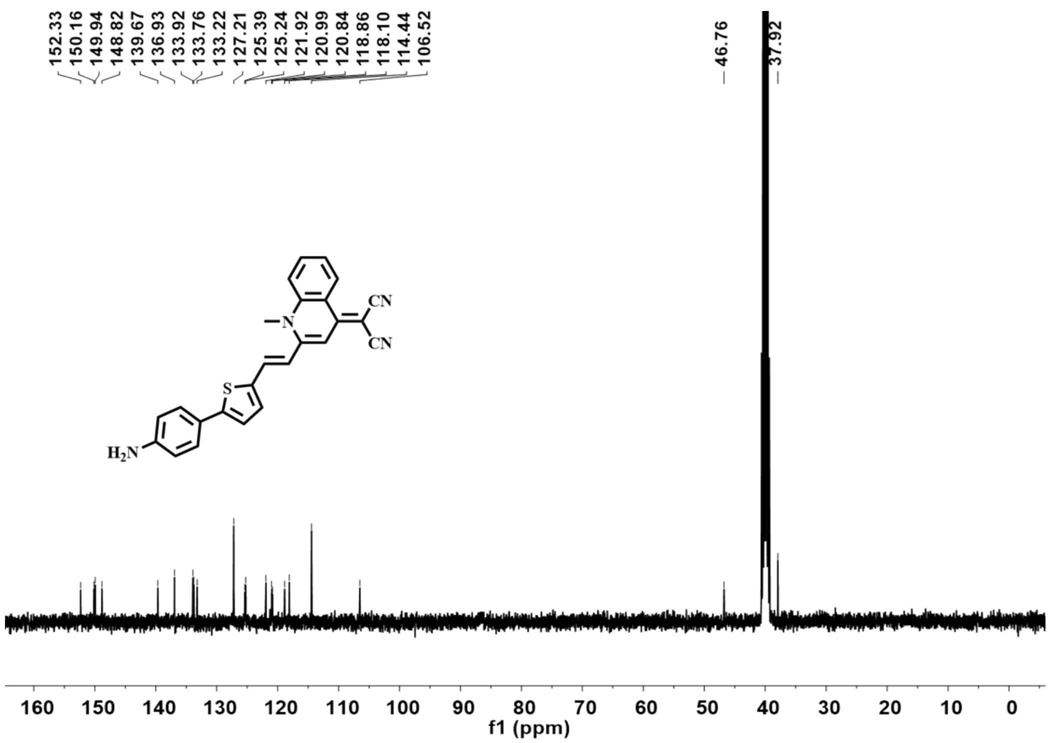

Fig. S18. ^{13}C NMR spectrum (100 MHz) of compound **2** in DMSO-d_6

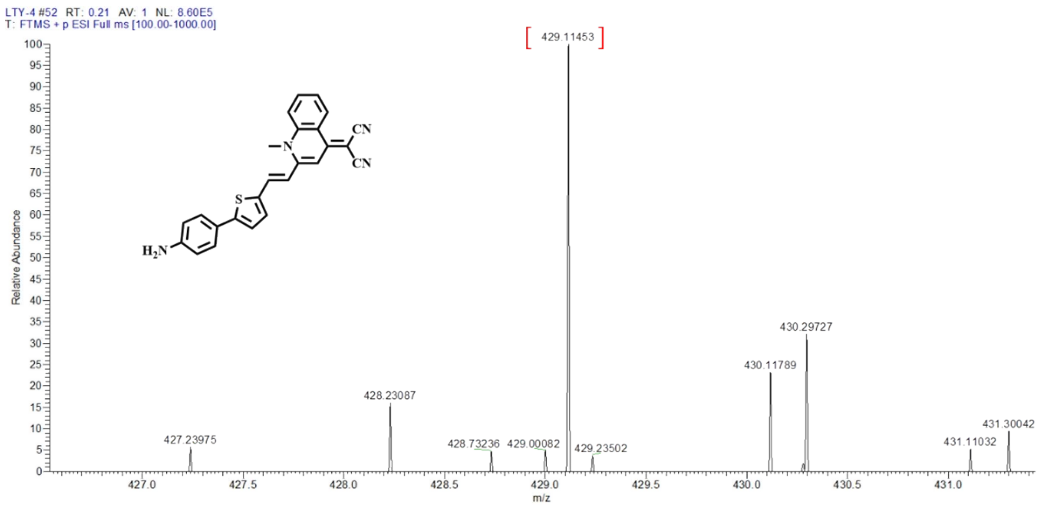

Fig. S19. HR-MS spectrum of compound **2**

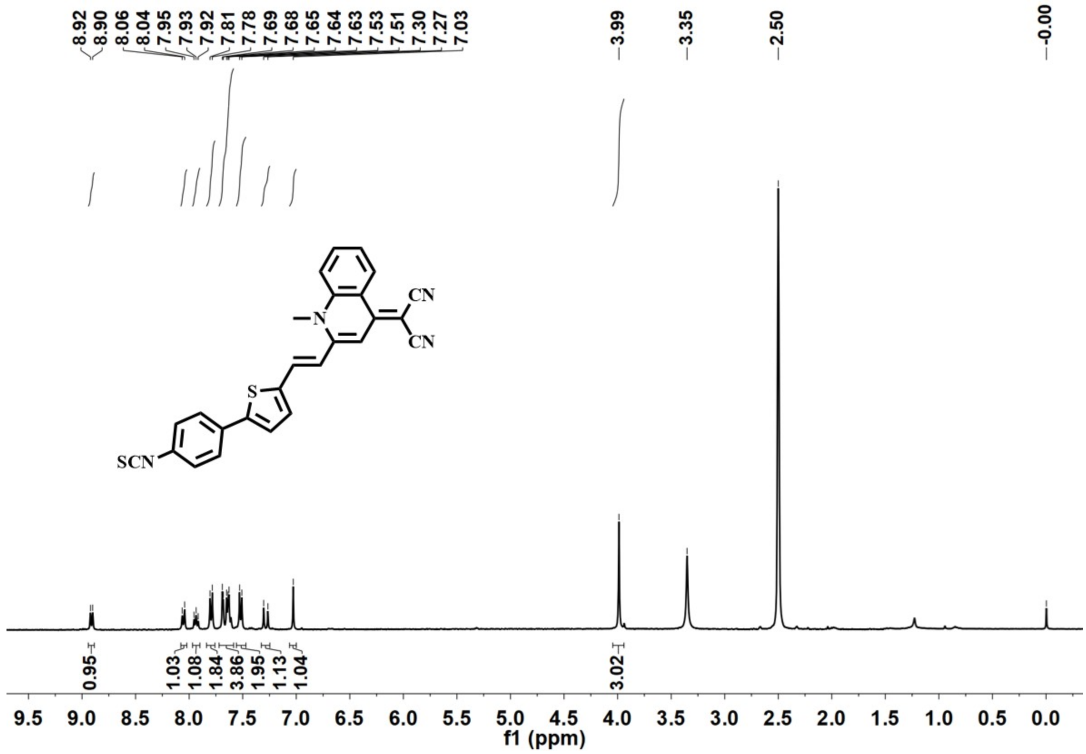

Fig. S20. ^{1}H NMR spectrum (400 MHz) of compound **1** in DMSO-d_6

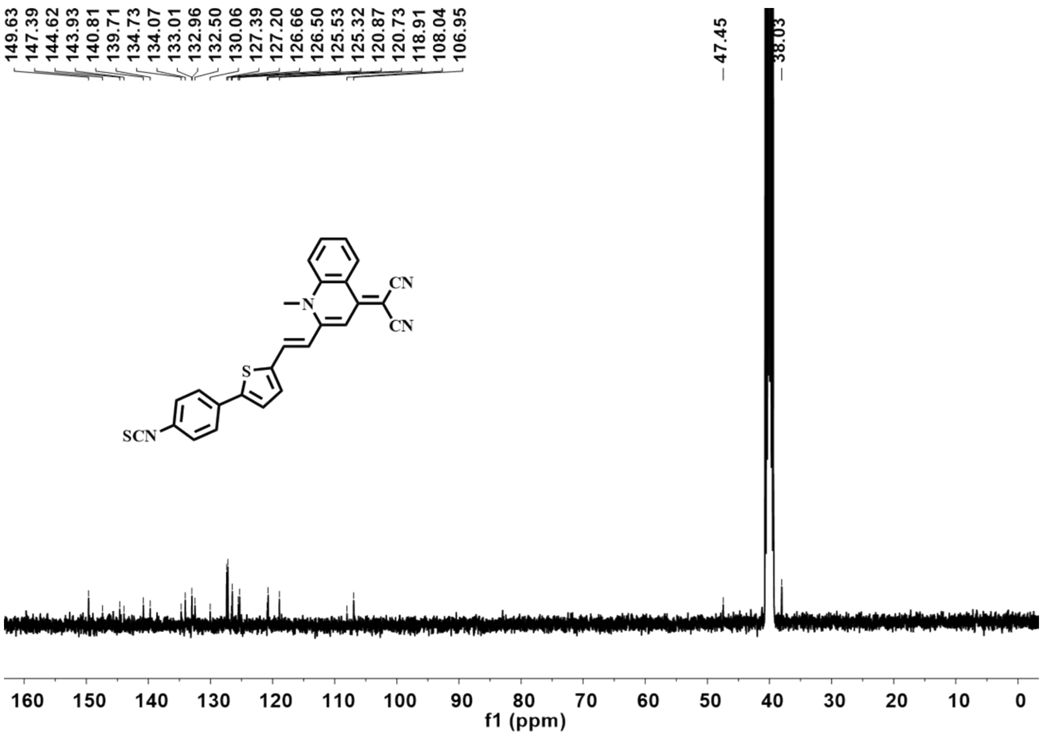

Fig. S21. ^{13}C NMR spectrum (100 MHz) of compound **1** in DMSO-d_6

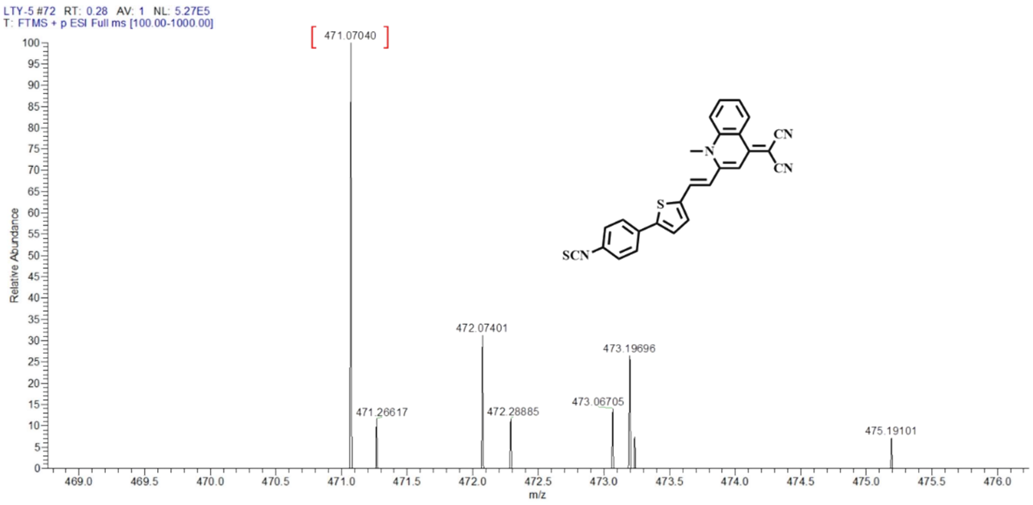

Fig. S22. HR-MS spectrum of compound **1**

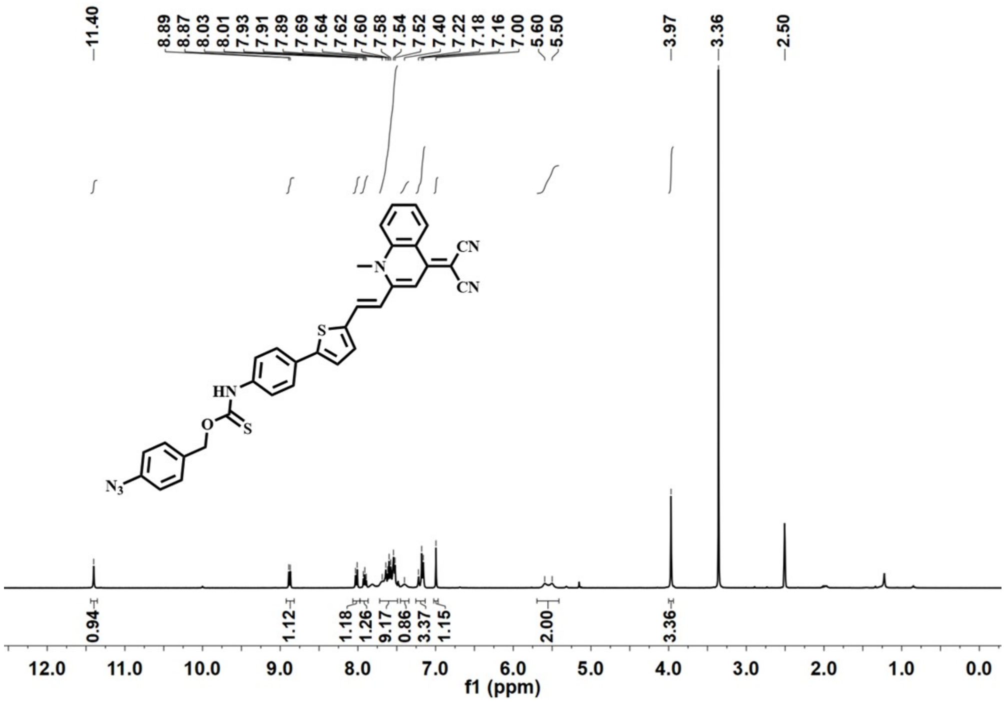

Fig. S23. ^{1}H NMR spectrum (400 MHz) of **HL-H$_2$S** in DMSO-d_6

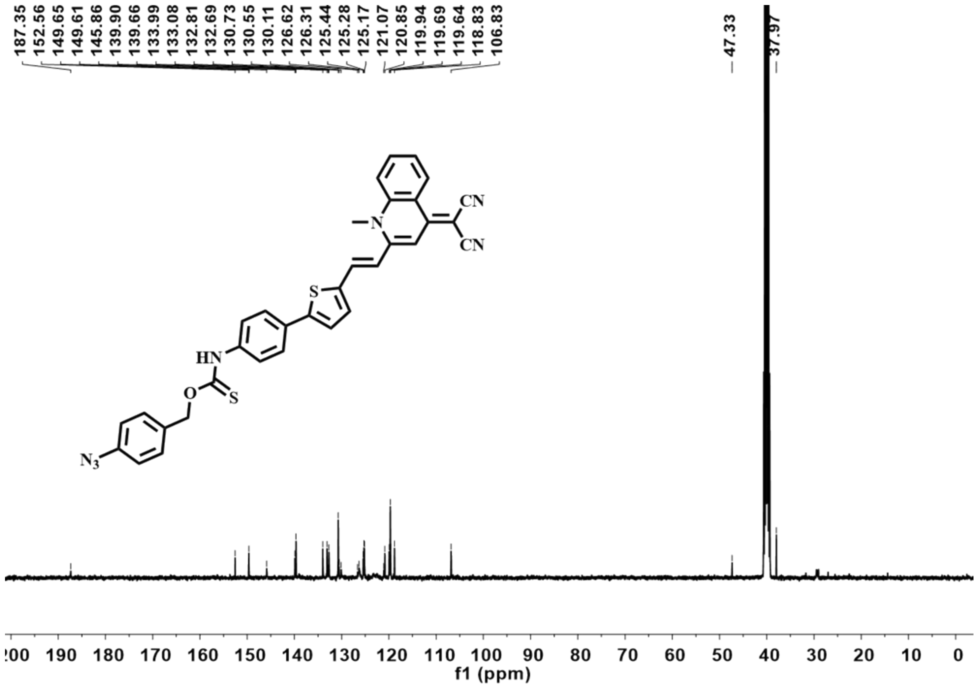

Fig. S24. ^{13}C NMR spectrum (100 MHz) of **HL-H$_2$S** in DMSO-d_6

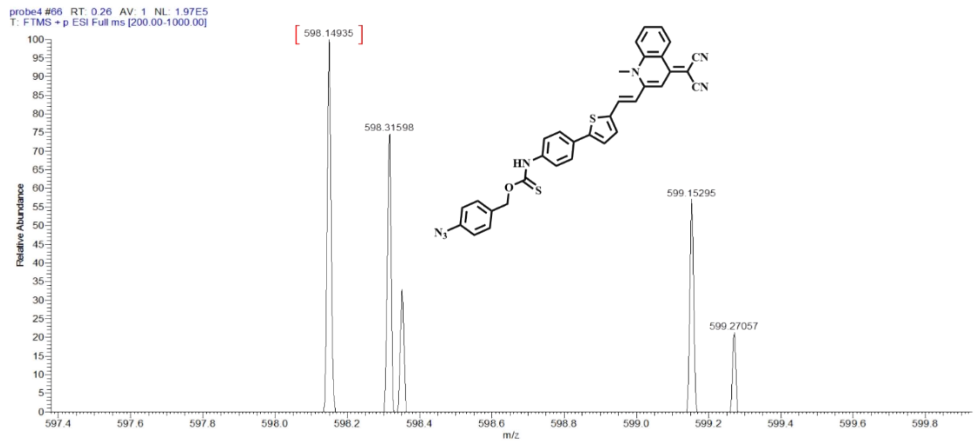

Fig. S25. HR-MS spectrum of **HL-H_2S**
